# Amino acids activate mTORC1 to release roe deer embryos from decelerated proliferation during diapause

**DOI:** 10.1073/pnas.2100500118

**Published:** 2021-08-27

**Authors:** Vera A. van der Weijden, Jochen T. Bick, Stefan Bauersachs, Anna B. Rüegg, Thomas B. Hildebrandt, Frank Goeritz, Katarina Jewgenow, Pieter Giesbertz, Hannelore Daniel, Emilie Derisoud, Pascale Chavatte-Palmer, Rupert M. Bruckmaier, Barbara Drews, Susanne E. Ulbrich

**Affiliations:** ^a^Animal Physiology, Institute of Agricultural Sciences, Eidgenössische Technische Hochschule Zurich, 8092 Zurich, Switzerland;; ^b^Functional Genomics Group, Institute of Veterinary Anatomy, University of Zurich, CH-8315 Lindau, Switzerland;; ^c^Reproduction Management, Leibniz Institute for Zoo and Wildlife Research, 10315 Berlin, Germany;; ^d^Reproduction Biology, Leibniz Institute for Zoo and Wildlife Research, 10315 Berlin, Germany;; ^e^Nutritional Physiology, Technische Universität München, 85354 Freising, Germany;; ^f^Université Paris-Saclay, Université Versailles-Saint Quentin, INRAE, Biology of Reproduction, Environment, Epigenetic and Development (BREED), 78350, Jouy-en-Josas, France;; ^g^Ecole Nationale Vétérinaire d’Alfort, Biology of Reproduction, Environment, Epigenetic and Development (BREED), 94700, Maisons-Alfort, France;; ^h^Veterinary Physiology, Vetsuisse Faculty, University of Bern, 3001 Bern, Switzerland

**Keywords:** embryonic diapause, European roe deer (*Capreolus capreolus*), embryo development

## Abstract

Embryonic diapause in mammals leads to a reversible developmental arrest. While completely halted in many species, European roe deer (*Capreolus capreolus*) embryos display a continuous deceleration of proliferation. During a 4-mo period, the cell doubling time is 2 to 3 wk. During this period, the preimplantation blastocyst reaches a diameter of 4 mm, after which it resumes a fast developmental pace to subsequently implant. The mechanisms regulating this notable deceleration and reacceleration upon developmental resumption are unclear. We propose that amino acids of maternal origin drive the embryonic developmental pace. A pronounced change in the abundance of uterine fluid mTORC1-activating amino acids coincided with an increase in embryonic mTORC1 activity prior to the resumption of development. Concurrently, genes related to the glycolytic and phosphate pentose pathway, the TCA cycle, and one carbon metabolism were up-regulated. Furthermore, the uterine luminal epithelial transcriptome indicated increased estradiol-17β signaling, which likely regulates the endometrial secretions adapting to the embryonic needs. While mTORC1 was predicted to be inactive during diapause, the residual embryonic mTORC2 activity may indicate its involvement in maintaining the low yet continuous proliferation rate during diapause. Collectively, we emphasize the role of nutrient signaling in preimplantation embryo development. We propose selective mTORC1 inhibition via uterine catecholestrogens and let-7 as a mechanism regulating slow stem cell cycle progression.

## Significance

In mammals, embryo development can halt at the hatched blastocyst stage. Uniquely, proliferation of diapausing embryonic roe deer cells decelerates to a doubling time of 2 to 3 wk over a period of 4 mo. We highlight nutrient sensing as an important factor regulating embryonic developmental pace. The resumption of embryo development is characterized by an increase in uterine fluid mTORC1-activating amino acids, embryonic mTORC1 activity, and expression of metabolism and cell cycle genes. We propose selective mTORC1 inhibition via reduced estrogen signaling and high let-7 levels as mechanisms for slow cell cycle progression. We hypothesize that it is the lack of embryonic mTORC2 inhibition during embryonic diapause in the roe deer that enables the continuous decelerated rate of proliferation.

Diapause in mammals refers to a reproductive strategy in which the embryo’s development is temporarily and reversibly halted (1). This physiological phenomenon takes place in more than 130 species of mammals (1). More than 150 y ago, Bischoff discovered embryonic diapause in European roe deer (*Capreolus capreolus*) (2). In this species, breeding takes place in July/August, yet fawns are born in May of the following year. Bischoff observed that the hatched blastocyst, with a size of up to 4 mm, rests 4 to 5 mo in the uterus until it resumes development and implants in late December/January (3). The resumption of embryo development at a rapid rate marks the end of diapause and is characterized by the morphological transition from a round to a lemon-shaped blastocyst at the initiation of embryo elongation. The latter is a common feature of all ungulates preceding implantation. Different from other known species exhibiting diapause, roe deer and nine-banded armadillo embryos continuously increase in size and cell number over the course of diapause (4–6). In the diapausing roe deer embryo, less than 10% of cells stain positive for the proliferation marker Ki67, and the fraction of Ki67-positive cells is slightly lower in the trophectoderm (TE) than in the inner cell mass (ICM) (7). The doubling time of the number of cells is estimated to be 18 to 21 d for TE cells and 21 to 26 d for the ICM cells (7). The underlying molecular mechanism regulating the decelerated developmental pace remains largely unknown.

Embryonic diapause has been studied in mice, tammar wallabies, spotted skunks, and mink. In these species, embryonic development comes to a full halt. In mice, diapause occurs either as facultative during lactation (8), or is reversibly inducible by ovariectomy and progesterone replacement (9). The inhibition of cell proliferation gradually proceeds from the TE to the ICM cells until it comes to a complete arrest (10). The dormant murine embryo displays a transcriptional state, which differs from the transcription profile found in day 3.5 ICM or reactivated epiblast cells (11). Components of the mammalian target of rapamycin (mTOR) pathway are reduced in the epiblast during diapause (12). The chemical inhibition of both mTOR complexes, that is, mTORC1 and mTORC2 (13), induces embryonic diapause in mice for up to 30 d (14). In carnivores, obligate diapause is most commonly regulated by seasonal cues. In mink, prolactin-mediated escape from diapause associated with an increasing photoperiod is characterized by a surge in cell proliferation by day 3 and an increase in embryonic size by day 5, after reactivation (15). In both mice and mink, the inhibition of intrauterine polyamine synthesis under conditions of low progesterone availability reversibly induces embryonic diapause (16–18).

During the preimplantation period, the maternal recognition of embryonic development results in cell type–specific endometrial gene expression changes (19, 20) and coincides with morphological changes of the embryo (21). A transcriptome analysis of reactivated versus diapausing mink uteri revealed an enriched up-regulation in canonical pathways such as protein folding, innate immune response, and the regulation of cell proliferation (22). In the spotted skunk, the expression of leukemia inhibitory factor coincides with the termination of embryonic diapause (23). The current consensus is that the uterus plays the decisive role in governing diapause and embryonic developmental pace in a cell type–specific manner either via direct signaling or via altering the composition of the uterine fluid.

The uterine fluid presents a complex mixture of embryonically and maternally secreted components including proteins, amino acids (AA), nutrients, ions, and metabolites (24–26). The absence of uterine glands and their secretions in sheep resulted in the failure of the embryo to develop past the blastocyst stage (27). An interspecies embryo transfer experiment, in which sheep embryos were transferred to the mouse uterus, indicated that the murine uterine environment during diapause was able to temporarily induce diapause in a species not featuring diapause (28). Thus, the distinct composition of the uterine microenvironment appears to affect developmental pace through common mechanisms that are partly conserved across species. We have previously demonstrated that the roe deer uterine fluid proteome presents an environment with high detoxification potential during diapause and that embryo elongation is characterized by the presence of cell proliferation–supporting proteins (29).

Here, we set out to identify the molecular regulatory mechanisms that regulate roe deer embryonic developmental pace by employing a holistic data integration approach. To this end, we first conducted de novo sequencing of the roe deer transcriptome. Extensive field sampling of roe deer, bagged by hunters, allowed us to collect embryos, uterine tissue, plasma, and uterine fluid from 283 female roe deer across the preimplantation period. Additionally, in vivo–developed embryos 13 d post-mating were flushed, and uterine samples were collected from captive roe deer. We assessed the impact of the uterine environment on the embryo by combining the transcriptomics data from both laser-capture microdissected luminal epithelial (LE) cells and single embryos with the uterine fluid AA profiles. Our approach sought to identify conserved molecular pathways responsible for regulating cell proliferation in embryos. We found that changes in the uterine microenvironment correlated with the activation of mTORC1 and were related to the observed increase in cell proliferation after the resumption of embryo development.

## Results

### Physiology of Decelerated Developmental Pace.

**Fig. 1.**
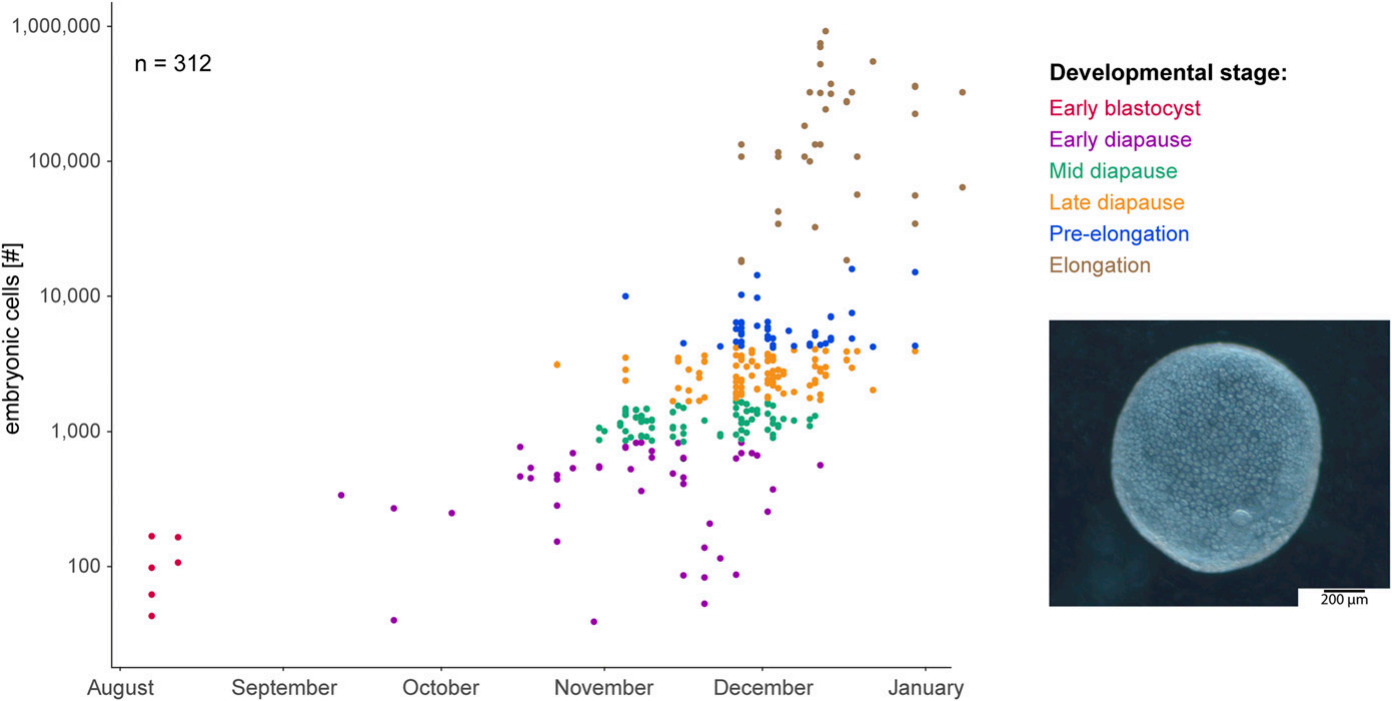
Developmental progression of preimplantation roe deer embryos from August to January with an example of an expanded hatched blastocyst in November. The embryonic number of cells were determined based on the embryonic genomic DNA content, and a doubling time of 2 to 3 wk in the number of cells was estimated prior to elongation. Dataset overlaps with previously published data (5).

Comparing the roe deer embryos collected during field sampling, we discerned a continuous increase in the number of cells based on the date of collection during the period of diapause. We divided the embryos into different stages, that is, early blastocyst obtained from in vivo flushings, and based on cell number, early, mid, and late diapause, pre-elongation, and elongation (Fig. 1). The temporal comparisons of the total cells per embryo suggest that the embryonic cells divided every 2 to 3 wk prior to elongation and every 1 to 4 d post-elongation.

### Embryo Elongation Coincides with Transcriptome Changes.

To investigate the molecular signaling pathways involved in the regulation of embryonic diapause, we assembled 63,935 sequenced transcripts to a roe deer transcriptome (*SI Appendix*, *Supplementary Information 1*). The annotated transcriptome consisted of 33,980 ortholog transcripts including 15,053 human unique transcripts hits and 14,892 different bovine unique transcripts hits (*SI Appendix*, *Supplementary Information 1*).

**Fig. 2.**
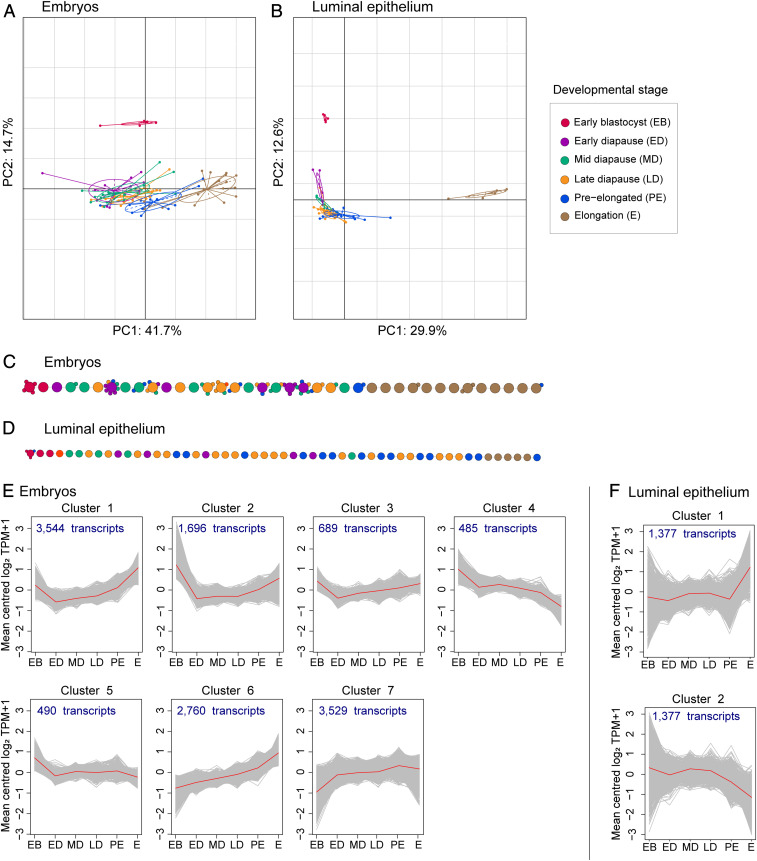
(*A* and *B*) Principal component–based between-group analyses for the embryos (*A*) and luminal epithelium (*B*). Colors indicate the defined developmental stages. Early blastocysts form a separate cluster, embryos during diapause display a developmental-stage specific transcriptome profile, which, in parts, overlap, and the elongated embryos form a separate cluster. (*C* and *D*) The pseudotime backbone derived from the CellTree algorithm (68) of single embryos (*C*) and luminal epithelium (*D*). Each circle represents a sample and the colors indicate the assigned developmental stage based on the number of embryonic cells. Samples with largely similar transcriptome profiles are clustered together around one main sample (smaller circles attached to each big circle), while samples with less similar profiles are placed next to each other (big circles), connected by a backbone. (*E* and *F*) Transcriptome dynamics displayed by a self-organizing tree algorithm (SOTA) of the differentially abundant transcripts as calculated with the ImpulseDE2 algorithm (69) with mean centered log2 TPM+1 at predefined developmental stages including early blastocysts (EB), early (ED), mid (MD), and late diapause (LD), preelongated (PE), and elongated embryos (E) for the embryos (*E*) and for the LE (*F*). The embryonic and luminal epithelial transcriptome SOTA clusters are derived by unsupervised clustering, and derived clusters represent a group of transcripts with similar changes of the expressed transcripts.

To identify molecular signaling pathways related to the control of embryonic developmental pace, we investigated the transcriptome changes in 87 single roe deer embryos and 56 laser-capture microdissected endometrial LE samples (*SI Appendix*, *Supplementary Information 1* and *2*). The LE RNA sequencing (RNA-seq) data showed a high degree of similarity to qPCR data (Dataset S4). The morphological transition from a blastocyst to an elongated embryo coincided with major transcriptome changes (Fig. 2*A*). Similarly, transcriptome changes in the LE coincided with embryo elongation (Fig. 2*B*). The defined developmental stages prior to elongation overlapped in the principal component analyses (PCA) (Fig. 2 *A* and *B*). By performing pseudotime analyses, we observed a high degree of transcriptional similarity among early blastocysts and among elongated embryos (Fig. 2 *C* and *D*). Both embryos and LE of all diapause stages displayed transcriptional similarities, indicated by their overlap in the PCA plot. However, there was quite some variance between different samples from the same developmental stage. This can be appreciated by the heterogeneous dispersion of these samples between the two ends of the pseudotime backbone tree (Fig. 2 *C* and *D*). By conducting time-course analyses, a total of 13,193 differentially expressed transcripts (DET) were identified in the embryos, while only 2,754 DET were identified in the LE. The embryonic transcriptional profile indicated an up-regulation of transcripts in six out of seven clusters (Fig. 2*E*). In the LE, transcript expression changes were absent during diapause but coincided with embryo elongation (Fig. 2*F*).

### Embryonic Transcriptome Profile.

To explore the biological functions of the DET in the embryos, the Qiagen Ingenuity Pathway Analysis (IPA) was deployed. Five of the 174 embryonically enriched canonical pathways had a negative enrichment at elongation compared to the earlier developmental stages (*SI Appendix*, Fig. S9 and Dataset S1). Genes in these five canonical pathways are related to cell cycle control. The transcriptional changes observed in the embryos were in silico predicted to be positively regulated by growth factors including fibroblast growth factors, vascular endothelial growth factor, hepatocyte growth factor, insulin-like growth factor 1 (IGF1), endothelial growth factor, and platelet-derived growth factor, the transcription factor CREB1, and E2, while they were in silico predicted to be negatively regulated by the microRNA let-7 and let-7a-5p. In fact, a whole group of microRNA was in silico predicted to be down-regulated at elongation. This group includes mir-1, miR-1–3p, miR-103–3p, mir-122, miR-122–5p, miR-124–3p, miR-125b-5p, miR-145–5p, mir-15, miR-155–5p, miR-16–5p, mir-17, miR-17–5p, mir-183, mir-19, mir-194, miR-199a-3p, miR-199a-5p, mir-210, mir-22, miR-22–3p, miR-26a-5p, miR-291a-3p, miR-29b-3p, miR-30c-5p, mir-34, miR-34a-5p, miR-375–3p, and miR-483–3p. Most let-7 and let-7a-5p targets were up-regulated at elongation (*SI Appendix*, Fig. S10 and Dataset S1). Furthermore, we investigated the regulation of developmental pace by assessing the embryonic expression of previously defined cell cycle phase–specific genes (30). The embryos displayed an increased expression of G1/S and S phase–specific genes prior to elongation (*SI Appendix*, Fig. S11).

### Maternal Control of Embryonic Developmental Pace.

Given the maternal contribution to embryonic diapause known in other species, we examined the LE transcriptome changes. We did not observe any transcriptional changes during the period of diapause (Fig. 2*F*). Pathway enrichment analyses evidenced a preparation for implantation, indicated by the enrichment of remodeling of epithelial adherence junctions and integrin signaling (*SI Appendix*, Fig. S9 and Dataset S1). Genes related to E2 signaling were down-regulated during diapause yet displayed an increase in expression at elongation (*SI Appendix*, Fig. S9 and Dataset S1). Next, we aimed at identifying the external and/or hormonal cues regulating embryonic diapause and quantified plasma AA, IGF1, and insulin. Additionally, we investigated the uterine LE expression of CLOCK genes and genes under control of melatonin and prolactin. There was no change in plasma AA, IGF1, and insulin during the period of diapause or at elongation. Furthermore, the uterine LE changes did not display an alteration in the expression of any of the circadian-, melatonin- or prolactin-related genes prior to embryo elongation (*SI Appendix*, Fig. S12).

### Uterine Microenvironmental AA Changes Are Related to Embryonic mTORC1 Signaling.

**Fig. 3.**
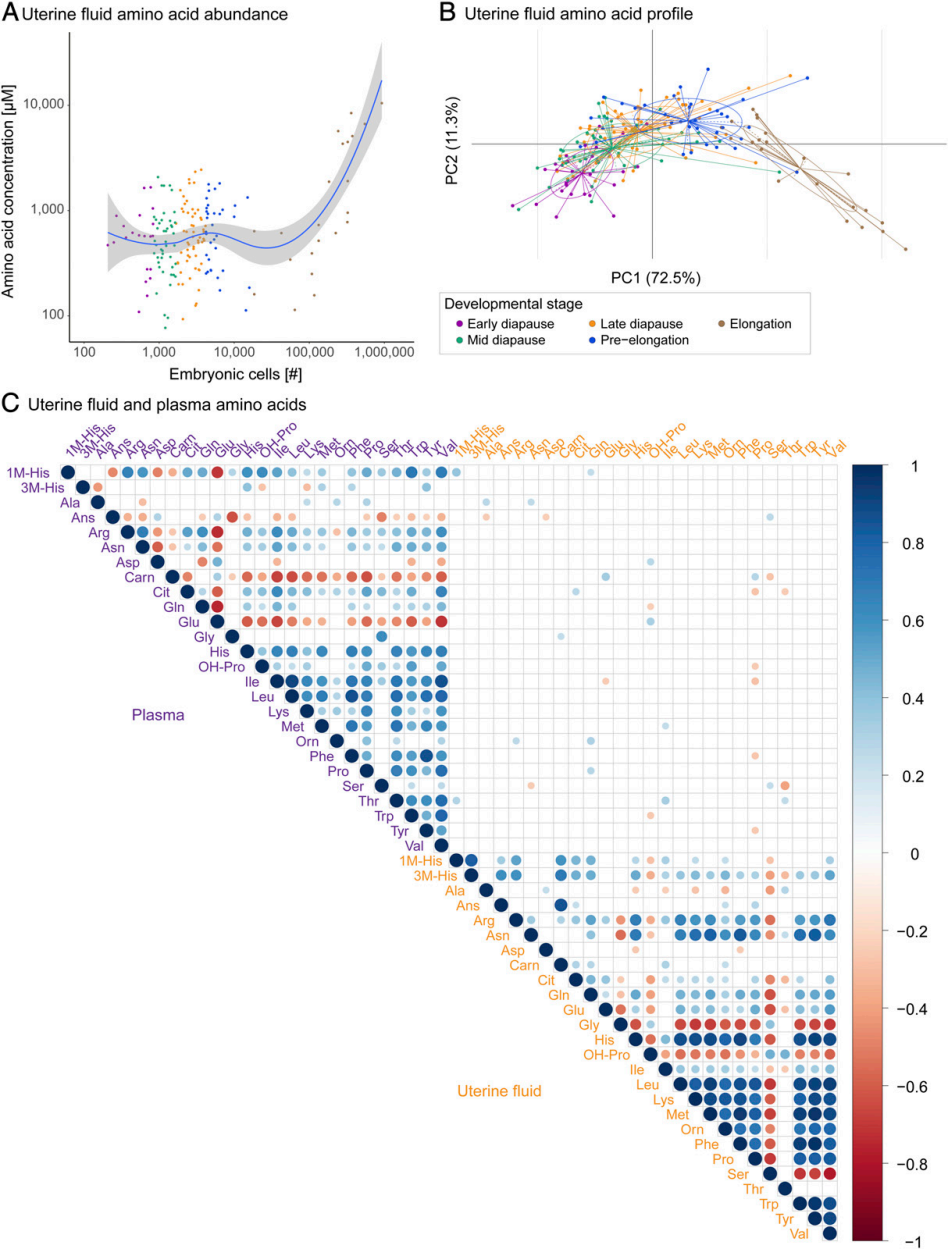
(*A*) Uterine fluid AA abundance against developmental progression defined as number of embryonic cells, displayed with a locally weighted scatterplot smoothing (Loess) regression. (*B*) Developmental stage–specific uterine fluid AA profile, displayed with a principal component based between-group analyses. ED: early diapause, MD: mid-diapause, LD: late diapause, PE: preelongation, and E: elongated. (*C*) Correlation plot of the uterine fluid and plasma AA, indicating a lack of correlation between uterine fluid and plasma AA. Statistically significant correlations are displayed (*P* < 0.05). The sizes of the dots indicate the degree of the correlation. The red dots indicate a negative correlation, while the blue dots indicate a positive correlation.

**Fig. 4.**
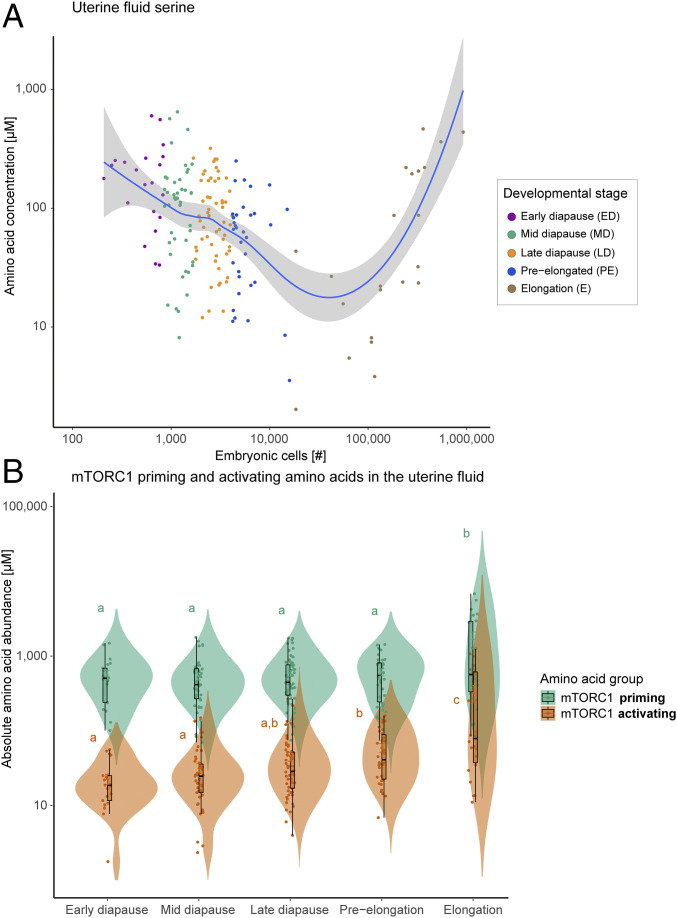
(*A*) Uterine fluid serine abundance against developmental progression defined as number of embryonic cells, displayed with a locally weighted scatterplot smoothing (Loess) regression. (*B*) Abundance of uterine fluid mTORC1 priming and activating AA per developmental stage. Priming is achieved by a group of AAs including l-asparagine, l-glutamine, l-threonine, l-arginine, l-glycine, l-proline, l-serine, l-alanine, and l-glutamic acid. The group of activating AAs includes l-leucine, l-methionine, l-isoleucine, and l-valine (31). An increase in mTORC1-activating AAs was evident from preelongation onward. The letters indicate statistically significant differences at *P* < 0.05.

The uterine fluid AA displayed an increase in absolute abundance with developmental progression (Fig. 3*A* and Dataset S2). The changes in AA composition were developmental stage-specific (Fig. 3*B*). They did not correlate with the abundance of maternal plasma AA (Fig. 3*C*). In contrast to the general trend of the increased abundance of AA, serine decreased with developmental progression (Fig. 4*A*). This was an ephemeral change, as its concentrations rebounded at elongation (Fig. 4*A*). From early diapause to preelongation, serine decreased 2.9-fold, while the concentration increased 1.6-fold from preelongation to elongation. From early diapause to preelongation, all quantified AAs displayed an increase of between 1.1- to 6.5-fold (with the exception of hydroxyproline with a fold change of only 0.7) (Dataset S2).

We investigated a possible role of AA in regulating cell proliferation. As mTORC1 signaling is driven by AA (31), we focused on the potential for AA that prime and activate this pathway. The cluster of AA that activates mTORC1 was substantially increased at preelongation, prior to the increase in the rate of embryonic developmental pace associated with elongation (Fig. 4*B*). All mTORC1-activating AAs increased from preelongation to elongation, that is, l-leucine, l-methionine, l-isoleucine, and l-valine increased by 5.4-, 7.0-, 5.5-, and 5.0-fold, respectively.

### Impact of Uterine Fluid AAs on the Embryo in Diapause.

**Fig. 5.**
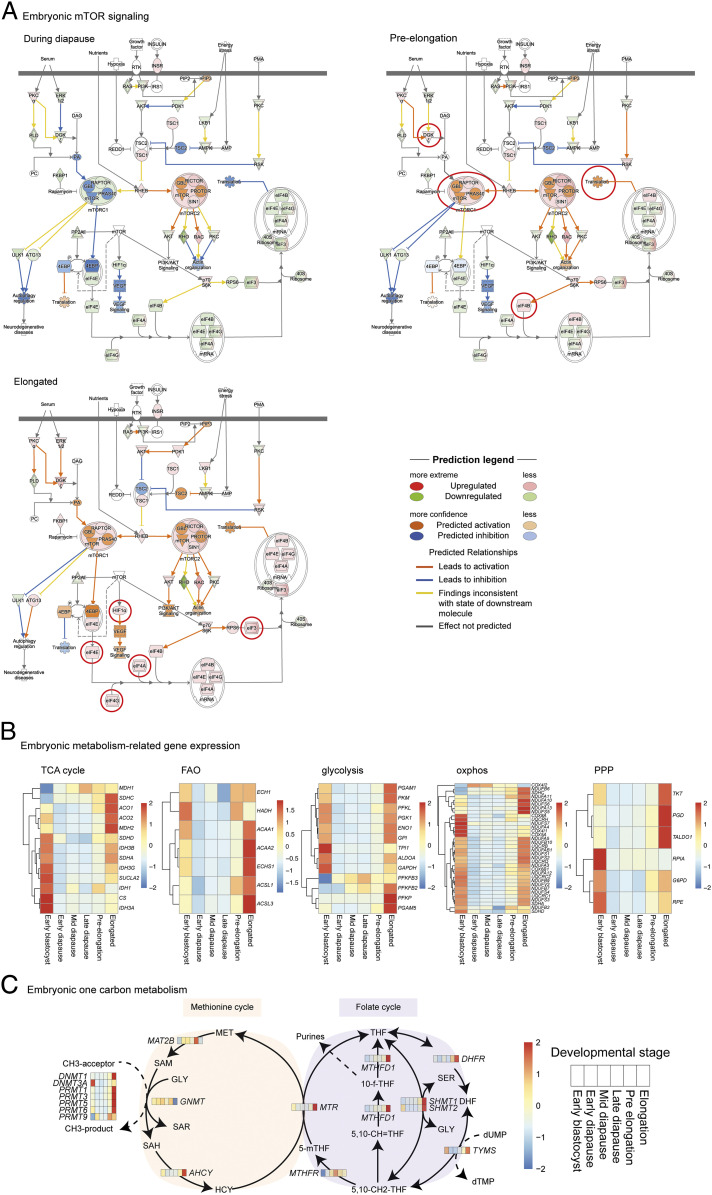
(*A*) Embryonic mTOR signaling during diapause, at preelongation, and during elongation. The pathway is derived from the Qiagen IPA software. All figures and data produced from IPA are available under an open-access CC-BY license for purposes of publication and are used with written permission. The mTORC1 is predicted to be inhibited during diapause and activated at preelongation. The red circles indicate transcriptional and predicted changes first observed at preelongation and elongation. (*B*) Embryonic expression of genes involved in the TCA cycle, fatty acid β-oxidation (FAO), glycolysis, oxidative phosphorylation (oxphos), and pentose phosphate pathway (PPP) displayed as *z*-scores of the fold changes. An increase in these metabolic pathways is observed from preelongation onwards. (*C*) Embryonic expression of genes involved in the one carbon metabolism. The embryonic gene expression and uterine fluid AA abundances are displayed in a heat map format in which each box represents one of the defined developmental stages, including the early blastocyst, early diapause, mid-diapause, late diapause, preelongation, and elongation stage.

We integrated the uterine fluid and embryo transcriptomics data to relate changes in uterine fluid mTORC1 priming and activating AA to the embryonic developmental pace. Based on the expression of genes related to the mTOR signaling pathway, the embryonic transcriptome indicated an increased mTORC1 signaling, starting at preelongation (Fig. 5*A*). In contrast, mTORC2 was predicted to be continuously activated during diapause and elongation. Functionally, the increase in mTORC1 signaling and rate of proliferation were accompanied by an increased expression of genes related to the glycolytic and phosphate pentose pathway, fatty acid β-oxidation, oxidative phosphorylation, the TCA cycle, and those characteristic of the one carbon metabolism/epigenetics (Fig. 5 *B* and *C*). Our data predict the activation of mTORC1 at preelongation, which is related to an increased metabolic activity of the embryo, supporting its proliferation by meeting the increase in energy demands, nucleotides, and DNA methyl donors.

## Discussion

Diapause in roe deer embryos is characterized by a remarkably slow continuous cell proliferation. In comparison, diapausing mouse, tammar wallaby, and mink embryos display an almost complete to full halt (32). While murine embryos can sustain in this diapause-like state for up to 30 d (14), the slowly proliferating roe deer embryo resides for an obligate 4 to 5 mo as free-floating embryo in the uterine fluid. During this period, the blastocyst grows to 4 mm without showing any signs of compromised development (4). In fact, the implantation rates are as high as 92% (4). The termination of diapause in the roe deer is morphologically easily assessable given the ungulate feature of embryo elongation. The molecular factors regulating embryonic developmental pace remain unknown. We hypothesized that resumption of a fast developmental pace is controlled by a molecular switch. This switch is either driven by the inherent genetic program of the embryo, for example, a threshold number of cells, or is a consequence of a biochemical cue of maternal origin. Given the feature of extended decelerated embryo development, the diapausing roe deer embryo is a unique model to study the regulation of proliferation in pluripotent cells.

Roe deer embryos were transcriptionally largely similar to each other during the entire period of embryonic diapause, while major changes appeared at embryo elongation. In line, single-cell RNA-seq of mouse embryos has previously revealed that early, mid-, and late blastocysts display transcriptional similarities (33). Like in other ungulates, major embryonic and endometrial transcriptome changes coincided with the rapid embryo elongation after the period of embryonic diapause (20, 34–36).

An important characteristic of preimplantation gestation is the presence of a distinct uterine fluid milieu. Its composition allows embryo survival to persist for an extended period. In species with protracted preimplantation development, this period, during which the embryo lacks maternal blood supply, takes on significance (4). Thus, the uterine fluid composition must play a crucial role in facilitating embryo development at a slow developmental pace. We observed major changes of uterine fluid AA concentrations over the course of diapause and prior to elongation. The uterine fluid AA changes occurred independent from the maternal plasma AA. Similar findings have previously been reported for fatty acids in the plasma, oviductal, and uterine fluid of the mare (37). Thus, the uterine microenvironment does not represent a mere transudate of the blood (38, 39) but is regulated separately. Specific maternal signals that regulate the pronounced uterine fluid changes during the period of diapause, preelongation, and elongation remain to be identified. Given the lack of maternal plasma IGF1 and insulin changes in nonfasted animals, we suspect that environmental changes such as food availability have little effect on the regulation of embryonic diapause. Despite the continuous presence of progesterone (40, 41), it remains intuitive to assume that the daylight length via melatonin may play a pivotal role in the regulation of the gonadotropic axis. We identified estrogens as an in silico–predicted upstream regulator of both the observed LE and embryonic transcriptome changes. In the embryo, both E2 and CREB1 downstream targets were more abundant from preelongation to elongation. E2-induced cell proliferation via CREB1 has previously been shown (42), thereby providing evidence for a potential regulatory role of E2. Previously, we have substantiated a lack of variation in both maternal plasma and endometrial steroid hormones, specifically E2 and P4, during this period of development of the roe deer embryo (40, 41). Thus, a possible explanation is that local intracellular E2 metabolism may occur as observed in mice (43). In mice, uterine conversion of E2 to catecholestrogens is required for embryo reactivation (44). The reactivation of embryo development via an E2 infusion has been associated with a rapid down-regulation of the embryonic microRNA let-7 (45). Thus, assessing intrauterine catecholestrogens is expected to shed further light on the conserved regulation of embryonic diapause and the uterine regulation of the fluid composition.

The distinct changes in uterine fluid AA were related to the change in developmental pace. Bovine, ovine, murine, and porcine embryos have an increasing energy demand with developmental progression (24, 46–53). This was reflected in the roe deer uterine fluid AA by an increase in abundance with developmental progression. Strikingly, the mTORC1-activating AAs displayed an increase from preelongation onward. mTORC1 is controlled by nutrients like glucose and AAs (13). In contrast to other ruminants (24, 54), serine decreased during diapause in the roe deer until precipitously increasing as the embryo elongates. Whether the reduction of the uterine fluid serine abundance at preelongation is a consequence of the embryonic uptake and metabolism or if it contributes to the continuous mTORC1 priming role of AA remains to be elucidated. Previously, the activation of mTORC1 has been shown to increase the de novo serine synthesis, thereby supporting de novo purine synthesis (55). The observed increase in embryonic mTORC1 signaling at elongation is in line with the observed increase in nucleotide synthesis via the pentose phosphate pathway and one carbon metabolism (56). In mice, both mTORC1 and mTORC2 must be switched off to establish a reversible state of embryonic diapause (14). Recently, the inhibition of mTORC1 and mTORC2 via the microRNA let-7 in maternally excreted extracellular vesicles has been evidenced in mice (57). In our dataset, embryonic transcriptome changes were in silico predicted to be under the upstream regulatory control of let-7. Like for let-7, a set of microRNA target genes was up-regulated at elongation, while these microRNA were predicted to be down-regulated at elongation. This suggests that microRNAs might play a role in the maintenance of embryonic diapause. Our data suggest a conserved regulation of embryonic diapause via uterine estrogens, let-7, and the embryonic mTOR pathway. We hypothesize that the lack of embryonic mTORC2 inhibition during embryonic diapause in the roe deer is related to the continuous decelerated rate of proliferation (7) and thus marks the difference to a complete halt as observed in mice.

**Fig. 6.**
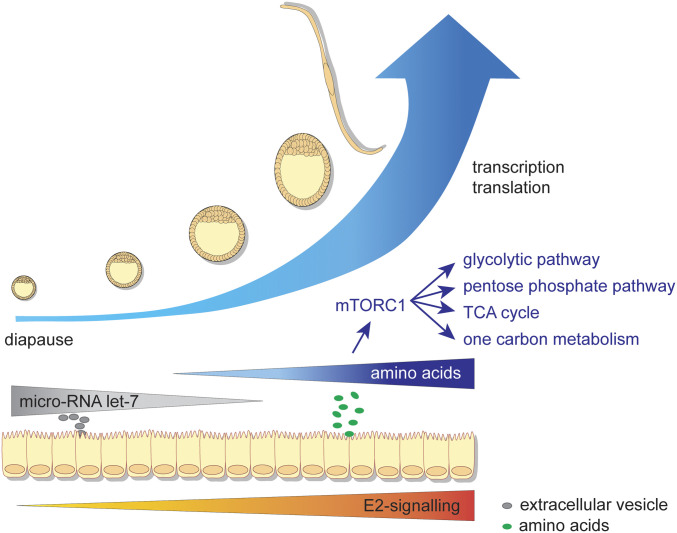
Proposed mechanism of the regulation of embryonic diapause in the roe deer. Endometrial E2 signaling is inhibited during embryonic diapause. The increase in the rate of proliferation in the embryo coincides with a decrease in microRNA let-7–driven gene expression in the embryos and a simultaneous increase in mTORC1 signaling. The increase in mTORC1 signaling is related to an increased expression of genes involved in the glycolytic and pentose phosphate pathway, the TCA cycle, and one carbon metabolism.

Conjointly, our findings provide a comprehensive molecular insight into the regulation of embryonic diapause in the roe deer since its discovery more than 150 y ago (Fig. 6). We hypothesize that during the phase of decelerated proliferation, mTORC2 remains active, while mTORC1 is inhibited. Prior to elongation, uterine fluid AAs activate mTORC1 and embryonic developmental pace, potentially regulated via uterine (catechol)estrogens and let-7. The fast rate of proliferation at reactivation is characterized by an increase in the embryonic glycolytic and pentose phosphate pathway, fatty acid β-oxidation, oxidative phosphorylation, the TCA cycle, and the one carbon metabolism. We anticipate our findings on the regulation of embryonic diapause in the roe deer to be pivotal for a more complex understanding of conserved embryonic developmental pace and cell proliferation regulatory mechanisms.

## Methods

### Sample Collection.

#### In vivo collection.

Endometrial biopsies and blastocysts were collected mid-August from a group of pregnant captive roe deer at day 13 post-mating by surgical embryo flushing (58). The two surgical access sites for inserting the 8 French Foley collection catheter into the distal part of each uterine horn provided sufficient exposed endometrium before the sites were closed with a single ligation. A modified pediatric heart biopsy forceps (short, customized model) was used to collect superficial endometrial samples (*n* = 6) from six females with an average weight of 0.0031 g ± 0.0009 g (Dataset S3). These samples were immediately snap frozen on aluminum foil in liquid nitrogen (−196 °C). Day 13 blastocysts (*n* = 6) were derived from captive roe deer by surgical in vivo embryo flushing as described in detail by Drews et al. (58) (Dataset S3). The animal experiment complied with German Animal Welfare law requirements and to the guidelines of the Internal Committee of Ethics and Animal Welfare of the Leibniz Institute for Zoo and Wildlife Research (approval no. 2347–25-2014) (58).

#### Field sampling.

All other samples collected between September to January were collected during regular huntings in 2015/2016, 2016/2017, and 2017/2018. The reproductive tract of a total 387 female roe deer, from which samples of 283 different does contributed to this study (Dataset S3), was obtained. Ethical approval was not required. The samples were collected after the animals were shot and kept on ice until further processing. Blood was sampled, and plasma was retrieved after centrifugation for 20 min at 2,000 × *g* at 4 °C of whole blood collected in EDTA tubes (Monovette). The uterus was dissected from residual connective tissue and flushed with 2.5 mL phosphate-buffered saline (PBS) to recover the embryos. The latter were visualized under a stereo microscope (Zeiss SteREO Discovery Microscope V8, 1:8 zoom rate equipped with an Olympus SC50 camera). The diameter of each embryo was determined, and after being washed in fresh PBS, the embryos were snap frozen in liquid N_2_. Endometrial cross-sections from each animal were taken, snap frozen in liquid N_2_ and stored at −80 °C. All samples were frozen within 8 h after the animal was shot. The retrieved uterine flushing volume was 76 ± 17% (mean ± SD). The fluid was centrifuged at 4 °C at 800 × *g* for 10 min. The supernatant was snap frozen within maximally 8 h after the animal was shot and stored at −80 °C until further analyses.

### Sample Selection.

Animals were included in the study if at least one embryo was recovered. Due to the field sampling procedure and limited available material from each animal relating to quality or quantity issues, different animals were used for the different analyses. The samples were treated independently for statistical analyses. As temporal changes during diapause and reactivation of embryo development have previously not been described in detail, the anticipated degree of change was unclear. A power calculation to estimate the sample size was therefore not conducted. Thus, it was decided to randomly select a subset of animals for each analysis, covering the entire preimplantation period to ensure a high density with regard to sampling date and embryonic developmental progression (*SI Appendix*, *Supplementary Information 2*). For the RNA-seq analysis, 56 endometrial samples from 56 different animals and 87 embryos from 60 different animals were used. For the uterine fluid AA profile, 180 animals were included in this study. A total of 75 plasma samples were included in the AA analysis, and 161 plasma samples were included in the IGF1 and insulin quantification. The samples used for each analysis can be found in Dataset S3.

### Laser-Capture Microdissection.

Endometrial cryosections of the in vivo– and field sampling–obtained samples were prepared for each tissue piece with a Leica CM 1950 Cryotome. The tissue was embedded in optimal cutting temperature compound (Biosystems, category no. 3801480S), and 10-µm sections were cut at a temperature of −25 °C and −20 °C for the cryo-chamber and specimen head. Sections were mounted on 1.0 PEN NF Membrane Slides (Zeiss, cat. no. 415190–9081-000) and stained with 1% (wt/vol) Cresyl violet acetate (Sigma Aldrich, cat. no. 86098–0) in 50% EtOH. The stained sections were stored at −80 °C until further processing. The intercaruncular luminal epithelium was collected with the Carl Zeiss Axio Observer with the AxioCam Cc1 camera, a LD Plan-NEOFLUAR 20× objective, the PALM MicroBeam laser, and the PALMRobo software (release 4.8, version 4.8.0.1). Samples were cut with an energy of 46 and a focus of 64. The laser pressure catapulting had an energy delta of 20 and a focus delta of 2. LE cells were collected in Adhesive Caps (Zeiss, cat. no. 415190–9191-000). All samples were stored at −80 °C until nucleic extraction.

### Isolation of Nucleic Acids, Quality Control, and RNA-Seq.

Embryonic DNA and total RNA were extracted with the Qiagen AllPrep DNA/RNA Micro Kit (Qiagen, cat. no. 80284) according to manufacturer’s instructions. The genomic DNA content was determined with the Promega Quantus and the QuantiFluor ONE dsDNA System (Promega, cat. no. E4870) according to manufacturer’s instructions. The total genomic DNA content was calculated and used to estimate the total number of cells (each cell containing 6 pg DNA). The cell number was used as developmental stage proxy for each embryo. The total RNA concentration and quality was assessed with the Agilent RNA 6000 Pico assay on the Agilent 2100 Bioanalyzer (Agilent Technologies). The RNA integrity number (RIN) was 5.0 ± 1.1 (average ± SD). There was no significant correlation between the time of the animal being shot to sample freezing and the RNA quality. Given the low RNA quality, the total RNA was subjected to library preparation with ribosomal RNA depletion with the Clontech Pico Input Mammalian Total RNA Kit according to manufacturer’s instructions. Eight libraries per lane were sequenced on the Illumina HiSeq 2500. The average sequencing depth was 48 million reads per sample.

The total RNA and DNA of the LE were extracted using the AllPrep DNA/RNA Micro Kit (Qiagen). After the isolation, the RNA integrity was determined with the Agilent 2100 Bioanalyzer RNA 6000 Pico Kit (Agilent, cat. no. G2939A). The samples displayed an RNA integrity of 6.8 ± 0.8 (mean ± SD). The total RNA was subjected to library preparation with the smart-seq2 protocol and sequenced on the NovaSeq (single-read 101 base pairs [bp]). The average sequencing depth was 20 million reads per sample.

### De Novo Transcriptome Assembly.

#### Data set preparation and sequencing.

Illumina TrueSeq RNA libraries were prepared starting from 100 ng total RNA from four embryos, 12 endometrial tissue samples, and a mixed tissue sample to capture as much transcripts as possible. From the four embryos used, one was an elongated embryo, and three were in the developmental stage of organogenesis. In addition, 12 endometrial samples were included. Three of those samples were collected during the pregnancy phase of the blastocyst stage (diapause), four during the elongated conceptus stage (after diapause), and four during the organogenesis stage (after implantation). Lastly, a mixed tissue sample including heart, lung, caruncles, elongate embryo, blastocyst, liver, kidney, spleen, ovary, oviduct, corpus luteum, and testicle was included. The data were sequenced on an Illumina HiSeq 2500 (126 bp paired-end reads) on two lanes at the Functional Genomic Centre Zurich (FGCZ). For the PacBio sequencing, three samples were selected including 1) a hatched blastocyst, 2) an elongated embryo, and 3) an endometrial sample. A large-scale PCR was performed for all samples before size selection: sample 1 with 18 cycles, sample 2 with 14 cycles, and sample 3 with 11 cycles. The size selection did not work for sample 1 because of the lack of sufficient material. For sample 2, three fractions were selected, including 1 to 2 kb, 2 to 3 kb, and >3 kb, while for sample 3, four fractions were selected, including 1 to 2 kb, 2 to 3 kb, 3 to 6 kb, and >6 kb.

#### RNA-seq analysis pipeline to retrieve the de novo assembly.

All FastQ files were analyzed on a local Galaxy server (59). All data sets were concatenated by tissue type. For the analysis, the “Best practices de novo with Trinity” (https://informatics.fas.harvard.edu/best-practices-for-de-novo-transcriptome-assembly-with-trinity.html) were followed. A detailed overview of the de novo assembly pipeline can be found the *SI Appendix*, *Supplementary Information 3*.

#### BLAST comparison to closely related species and human.

The complete transcriptome was BLASTed against all transcripts of the cattle genome (Bos taurus ARS-UCD1.2) and against all human transcripts (hg38, Homo sapiens p12). To that end, the current transcripts from the National Center for Biotechnology Information (NCBI; https://www.ncbi.nlm.nih.gov/assembly/GCF_002263795.1/, https://www.ncbi.nlm.nih.gov/assembly/GCF_000001405.38/) were downloaded and BLASTed with a nucleotide BLAST. The results were filtered on bitscore and query coverage (bitscore ≥ 100 and query_coverage ≥ 50%). In addition, the transcriptome was BLASTed with a nucleotide BLAST against the Ref-seq messenger RNA sequences to obtain a higher annotation rate, resulting in 15,277 unique gene symbols.

### Quantitative Liquid Chromatography–Tandem Mass Spectrometry Analysis of Uterine Fluid AAs.

Quantitative measurements of AA concentrations were performed using targeted liquid chromatography–tandem mass spectrometry (LC-MS/MS) based on previously described methods (60, 61). A detailed description can be found in *SI Appendix*, *Supplementary Information 3*.

### Quantitative LC-MS/MS Analysis of Plasma AAs.

For each sample, 50 µL plasma was mixed with 50 µL 10% 5-sulfosalicylic acid (SSA) with 2,500 µM NVal. The mixture was centrifuged for 4 min at 4 °C at 14,000 rpm. Of the supernatant, 10 µL was mixed with 70 µL borate buffer and 20 µL 2AMT. The mixture was incubated for 10 min at 50 °C, and 1/50 was injected in the ultra performance liquid chromatography for analysis with the Amino Acid Analysis MassTrak method (Waters). The quantified amounts in picomole were multiplied by 100 to compensate for the dilutions.

### Insulin and Insulin Growth Factor 1 Quantification.

Insulin and insulin-like growth factor 1 were quantified in plasma to identify any changes related to a change in nutritional status of the animals. The plasma insulin concentration of 161 animals was quantified with the PerkinElmer human AlphaLISA Insulin Detection Kit (product no.: AL350HV/C/F) as described previously by Robles et al. (62). A detailed description can be found in *SI Appendix*, *Supplementary Information 3*. The detection and quantification limits for the assay were 0.44 and 1.32 mUI/L, respectively. The inter- and intra-assay coefficient of variation were 15 and 10%, respectively.

For the same 161 animals, the plasma insulin-like growth factor 1 (IGF1) concentration was quantified with an immunoradiometric assay (ref. A15729, Beckman Coulter). The separation of IGF1 from IGF-binding proteins was performed by incubation and mixing 50 µL sample with 1 mL dissociation buffer (included in the assay kit). Intra- and inter-assay coefficients of variation were 5.6 and 8.3%, respectively.

### Bioinformatics and RNA-Seq Data Analysis.

Raw sequence reads (FastQ files) were analyzed on a locally installed Galaxy system (63). Basic read statistics and read quality was evaluated based on FastQC reports (64), and a MultiQC overview report of all samples was generated (65). Adaptors were clipped, sequences shorter than 50 bp were removed, and a low-quality end score of 30 was applied with the Trim Galore! tool. Duplicates were removed with the FastUniq tool (66), after which reads were mapped against the roe deer transcriptome with the Salmon tool (67). The count table was filtered with a counts per million cutoff of 3.0 and sample cutoff of 5. To gain insight into the dynamic changes, the CellTree algorithm was used to generate a pseudotime (68), and developmental stages were assigned and used to visualize the pseudotime. Six developmental stages were defined based on morphological characteristics of the embryo and the embryonic number of cells. The stages included the early blastocysts obtained from in vivo flushings, early (<833 cells per embryo), mid- (834 to 1,667 cells per embryo), and late diapause (1,668 to 4,167 cells per embryo), preelongation (4,168 to 16,667 cells per embryo prior to embryo elongation) and elongated embryos (>16,668 cells per embryo after embryo elongation). The SD filter was set to 1.5. The transcripts per million table was used with the ImpulseDE2 algorithm to identify DET in a time series–dependent manner (69). An adjusted *P* value of <0.05 was used. The roe deer DET were annotated against the human and bovine genome, and subsequent functional analysis was conducted on the annotated differentially expressed genes. A between-group analysis (BGA) was performed in R on mean-centered log_10_-normalized data (70). A self-organizing tree algorithm was used to cluster groups of transcripts according to their similarity in expression profiles (71). The functional analysis was conducted with the Qiagen IPA software.

### AAs Data Analyses and Statistical Analyses.

To guarantee a high coverage with regard to sampling date and developmental progression, all samples were selected randomly. For the uterine fluid analyses of AAs, 26 early diapause, 45 mid-diapause, 58 late diapause, 34 preelongation, and 22 elongated samples were analyzed. For the plasma AAs, 10 early diapause, 15 mid-diapause, 20 late diapause, 17 preelongation, and 10 elongated samples were analyzed. A BGA was performed in R on mean-centered log_10_-normalized data (70). Data were normalized against the sum of all AAs per sample (72), and normalized data were used for the correlation analysis (73). Absolute data were used for regression analyses in R (74). The regression type was set on locally weighted scatterplot smoothing (Loess) regression, allowing local fitting with the weighted least squares method.

## Supplementary Material

Supplementary File

Supplementary File

Supplementary File

Supplementary File

Supplementary File

## Data Availability

The data discussed in this publication have been deposited in NCBI's Gene Expression Omnibus (GEO) and are accessible through GEO Series Accession No. GSE158806 (75).
