# Supplementary material for: Amino acids activate mTORC1 to release roe deer embryos from decelerated proliferation during diapause
Source: Proc Natl Acad Sci U S A. 2021 Aug 27;118(35):e2100500118. doi: 10.1073/pnas.2100500118 (PMC8536382; doi:10.1073/pnas.2100500118)
Supplement: Supplementary File [file pnas.2100500118.sapp.pdf]

PNAS
www.pnas.org

Supplementary Information for

Amino acids activate mTORC1 to release roe deer embryos from decelerated proliferation during diapause.

Vera A. van der Weijden, Jochen T. Bick, Stefan Bauersachs, Anna B. Rüegg, Thomas B. Hildebrandt, Frank Goeritz, Katarina Jewgenow, Pieter Giesbertz, Hannelore Daniel, Emilie Derisoud, Pascale Chavatte-Palmer, Rupert M. Bruckmaier, Barbara Drews and Susanne E. Ulbrich

Corresponding author: Susanne E. Ulbrich
Email: seu@ethz.ch

This PDF file includes:

Supplementary information 1 to 3
Figures S1 to S12
Legends for Datasets S1 to S4
SI References

Other supplementary materials for this manuscript include the following:

Datasets S1 to S4

Supplementary Information 1

Transcriptome assembly results

All samples were analysed tissue-wise. The endometrium, embryo, and mixed tissue obtained by field sampling had ~407, ~267, and ~275 million reads after sequencing, respectively. After removing RNA-seq errors with Rcorrector, around 90% of the reads remained (Figure S1). The next tool to remove polymerase chain reaction (PCR) duplicates (FastUniq) filtered approximately 35% of reads out. The last tool, TrimGalore! that was used to improve the read quality, excluded around 10% of the remaining reads. Finally, ~223 million, ~144 million, and ~132 million paired-end sequences for endometrium, for the embryo, and the mixed tissue, respectively, (Figure S1) were used as the input for the Trinity *de novo* transcriptome assembler.

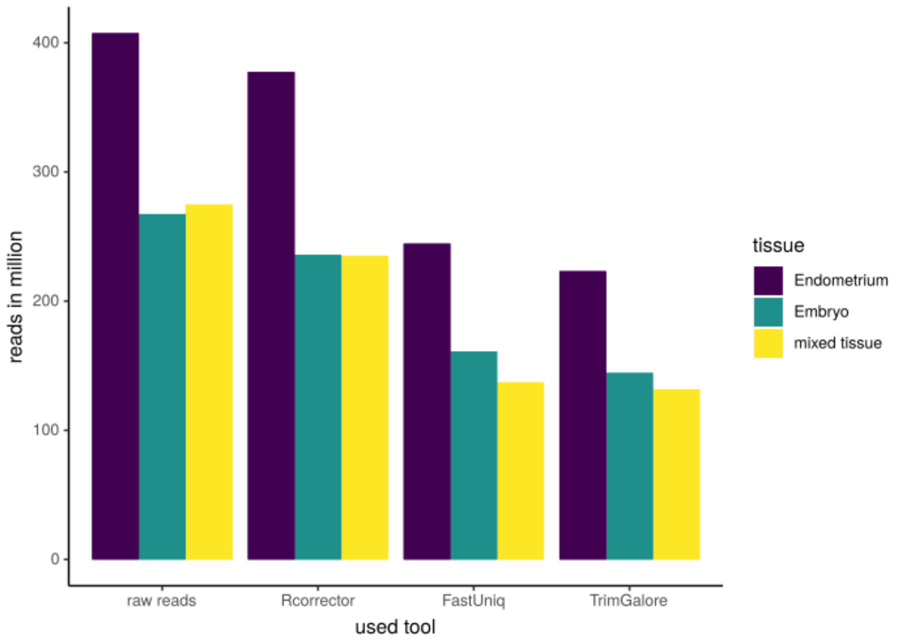

Figure S1: Number of paired-end reads after each filtering and optimizing process from raw FASTQ files to the final quality trimming with TrimGalore!

The resulting three assemblies had a number of detected transcripts between 659,734 (embryo), 660,087 (endometrium), and 1,040,742 (mixed tissue). After mapping all reads back to the assembly with salmon, a filtering on two transcripts per million (TPM) was performed. For the embryo tissue (EM), endometrium (EN) and mixed tissue (MT), 46,652 transcripts, 50,673, and 44,338 remained, respectively. By filtering on two TPM, we removed mainly short low abundant transcripts and possible artefacts stemming from the assembler. The transcripts of the three transcriptomes were also converted into peptides by Transdecoder, resulting in 10,159 peptides for EM, 13,430 for EN, 15,563 for MT, and 25,520 for the joined assembly (JA) (SI1 Table 1).

SI1 Table 1. Statistics for the four assemblies

assembly	N50	mean length [nt]	median length [nt]	max length [nt]	min length [nt]	# of transcripts TPM>2	mean TPM	median TPM	# of transcripts raw assembly	% of TPM>2	# of peptides
**embryo**	2008	1335	936	18,395	201	46,652	13.63	4.00	659,734	7.07	10,159
**endo-metrium**	2202	1494	1082	15,182	201	50,673	14.80	4.59	660,087	7.68	13,430
**mixed tissue**	2589	1747	1307	16,955	201	44,338	10.57	3.29	1,040,742	4.26	15,563
**joined**	2575	1628	1127	18,395	201	63,935	14.47	5.38	125,119	51.10	25,520

Additionally, all three assemblies and the JA were checked before and after TPM cut-off for quality, which is illustrated in Figure S2-5 (EM, EN, MT, and JA). First, we analysed the general distribution of the number of transcripts over the transcript frequency (TPM) (Figure S2-5, A and B). A) shows the distribution for TPM ranging from 0 to 1000 and B shows the range for lowly covered transcripts including transcripts ranging from 0 to 5 $\log_2$(TPM+1). The results in A) and B) show that most transcripts have a very low coverage between 0 and 1 $\log_2$(TPM+1). In all three assemblies, a high percentage (92% to 96%) of transcripts had a very low frequency below two TPM. The plots in C) show that these lowly expressed transcripts are mostly very short with a median length of ~400 bp. The plots in D) show different factors (see Methods section) that describe the assemblies for different possible TPM filter cut-offs. All four different assemblies (three individual and the joined assembly) show that filtering on two TPM keeps the longest transcript, and increases the median length, mean length and N50 of all assemblies. The joined assembly shows a different picture because it was constructed by joining the already TPM filtered individual assemblies. The TPM cut-off did not reduce the overall number of transcripts as much as in the other assemblies.

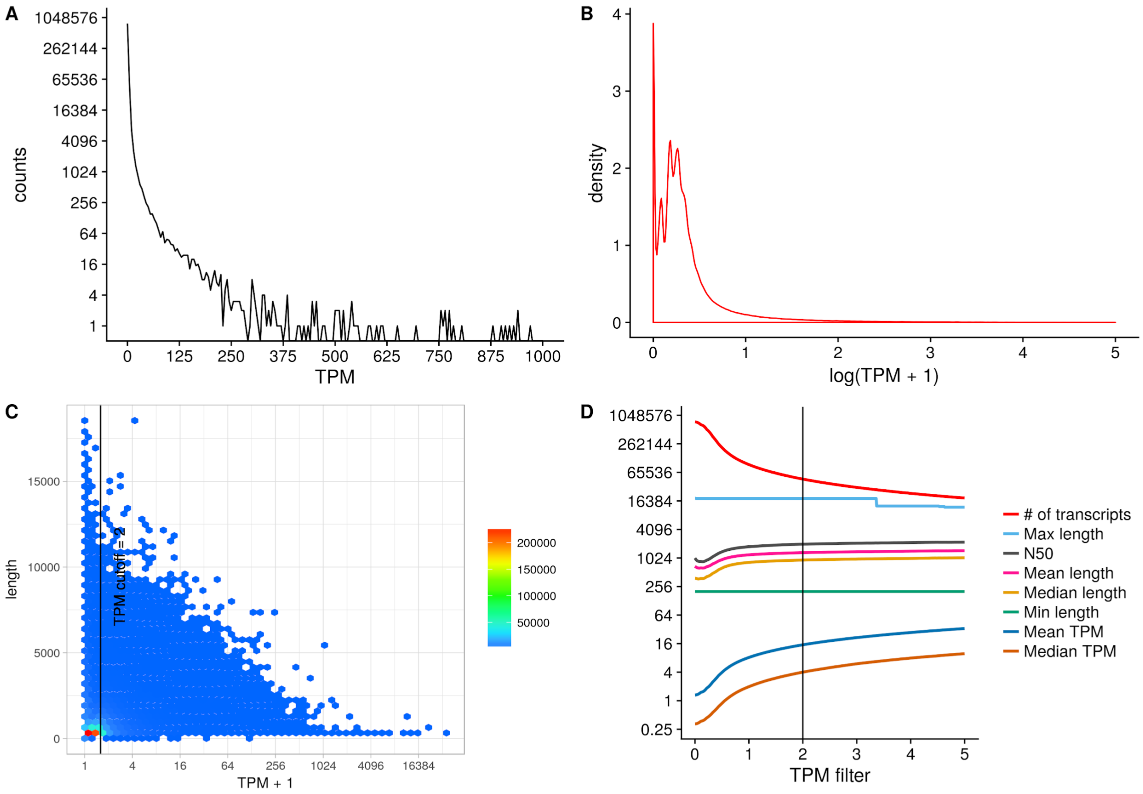

Figure S2. Assembly overview before filtering of the embryo. **A.** Frequency plot of the distribution of the TPMs ranging from 0 to 1000 with a bin-size of 5. **B.** Frequency plot of the distribution of the TPMs for lowly covered transcripts with a $\log_2$(TPM+1). **C.** Scatter plot of TPM + 1 versus sequence length, and **D.** Additional factors of the assembly for different TPM cut-offs ranging from no cut off to transcripts higher than 5 TPMs. Factors include N50, mean and median length of all transcripts, min and max length within the assembly, number of transcripts representing the assembly, and mean and median TPM of all transcripts.

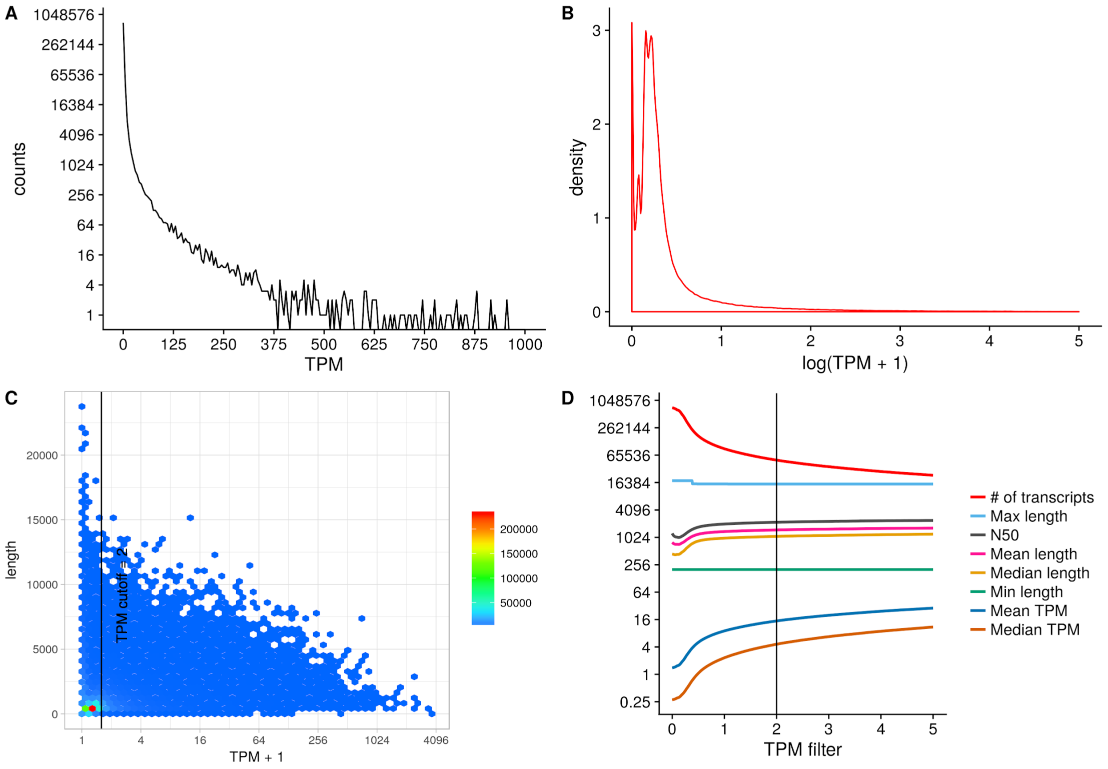

Figure S3. Assembly overview before filtering of the endometrium. **A.** Frequency plot of the distribution of the TPMs ranging from 0 to 1000 with a bin-size of 5. **B.** Frequency plot of the distribution of the TPMs for lowly covered transcripts with a $\log_2$(TPM+1). **C.** Scatter plot of TPM + 1 versus sequence length, and **D.** Additional factors of the assembly for different TPM cut-offs ranging from no cut off to transcripts higher than 5 TPMs. Factors include N50, mean and median length of all transcripts, min and max length within the assembly, number of transcripts representing the assembly, and mean and median TPM of all transcripts.

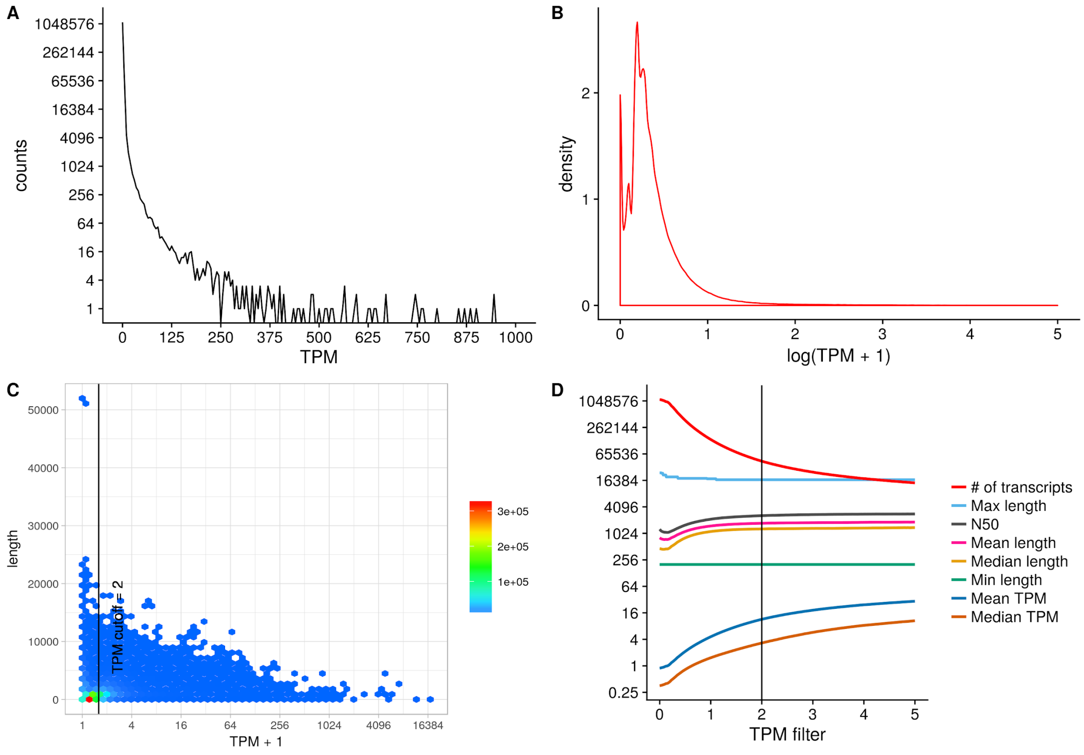

Figure S4. Assembly overview before filtering of the mixed tissue. **A.** Frequency plot of the distribution of the TPMs ranging from 0 to 1000 with a bin-size of 5. **B.** Frequency plot of the distribution of the TPMs for lowly covered transcripts with a $log_2(TPM+1)$. **C.** Scatter plot of TPM + 1 versus sequence length, and **D.** Additional factors of the assembly for different TPM cut-offs ranging from no cut off to transcripts higher than 5 TPMs. Factors include N50, mean and median length of all transcripts, min and max length within the assembly, number of transcripts representing the assembly, and mean and median TPM of all transcripts.

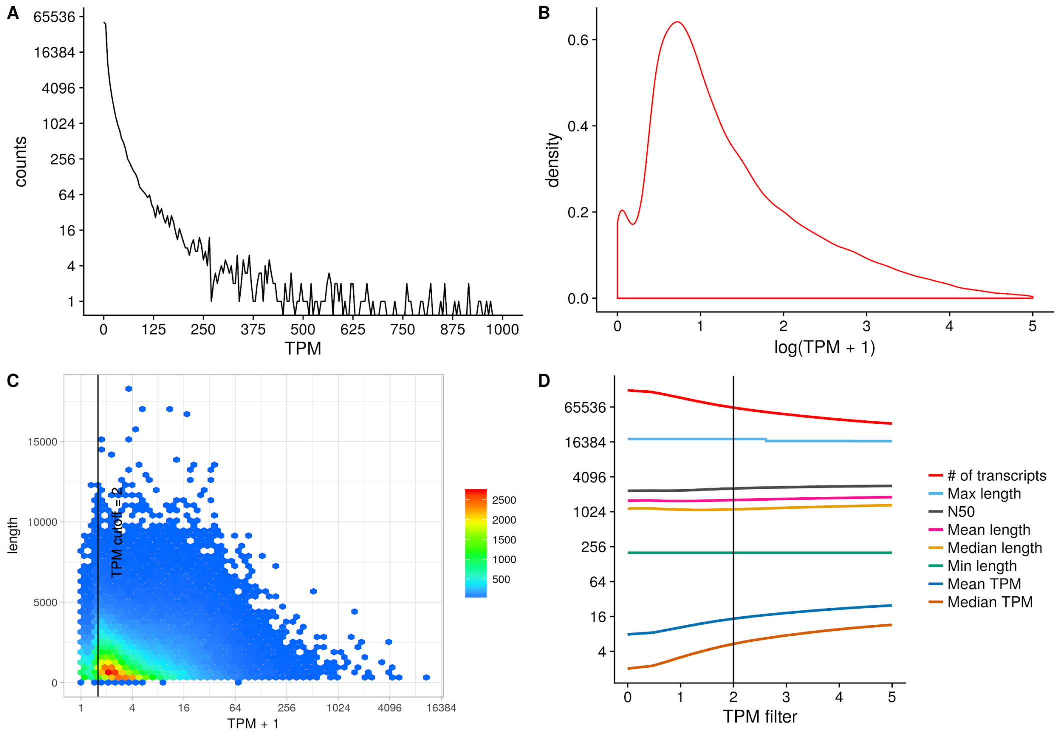

Figure S5. Joined EN, EM and MT. Assembly overview before filtering. **A.** Frequency plot of the distribution of the TPMs ranging from 0 to 1000 with a bin-size of 5. **B.** Frequency plot of the distribution of the TPMs for lowly covered transcripts with a log_2(TPM+1). **C.** Scatter plot of TPM + 1 versus sequence length, and **D.** Additional factors of the assembly for different TPM cut-offs ranging from no cut off to transcripts higher than 5 TPMs. Factors include N50, mean and median length of all transcripts, min and max length within the assembly, number of transcripts representing the assembly, and mean and median TPM of all transcripts.

PacBio long read assemblies

The resulting PacBio assemblies for elongated embryo and endometrium samples were statistically evaluated and then used for the Trinity assembler as a guide. All of the three assemblies had between ~51,000 and ~66,000 sequences (see SI1 Table 2). The N50, which is often used as a quality control of the assembly, was calculated and was between ~2,700 and ~3,700 nt. The assembly was also compared to the resulting filtered result of Trinity. Figure S6 shows that the PacBio assemblies alone represent approximately 50% to 60% of the resulting transcripts of the combined assembly approach (Illumina and PacBio reads).

SI1 Table 2. Assembly statistics of all three PacBio assemblies

sample	N50	mean length [nt]	median length [nt]	max length [nt]	min length [nt]	number of sequences
PacBio embryo	2761	2380.6	2426	20630	301	66067
PacBio endometrium	3730	3327.8	3177	23217	396	59597
PacBio merged embryo& endometrium	3469	3220.9	2971	22702	456	51119

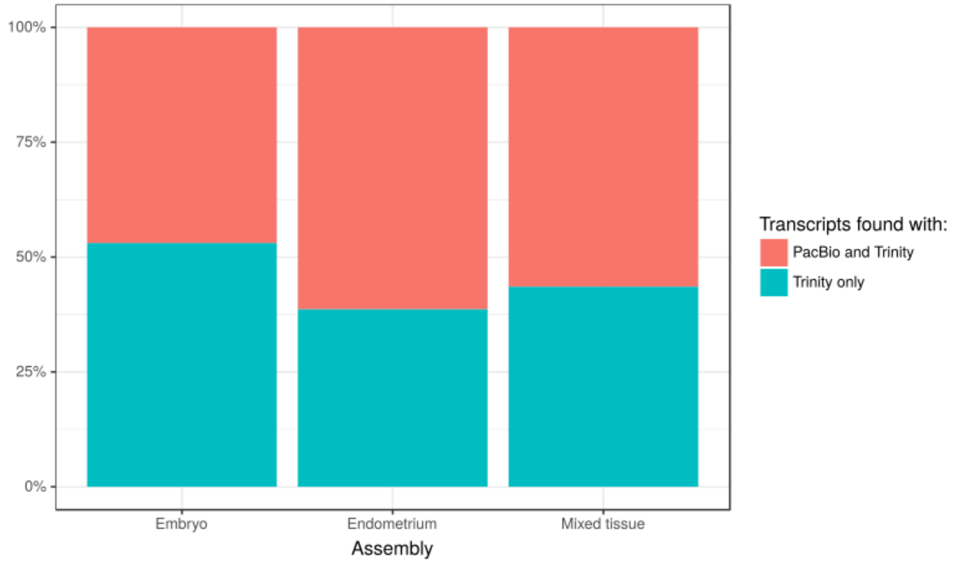

Assembly	Total counts	PacBio and Trinity	Trinity
Embryo	46652	21894	24758
Endometrium	50673	31076	19597
Mixed tissue	44338	25016	19322

Figure S6. BLASTn result of the three Trinity *de novo* transcriptome assemblies (EM, EN, and MT) versus the three *de novo* PacBio transcriptome assemblies.

BLASTn for all transcriptome assemblies against human and bovine transcriptome and proteome

To identify the assembled transcripts and corresponding genes by finding homologous sequences in related species, we used BLASTn versus the human and bovine transcriptome. For the joined transcriptome assembly, we found 33,980 orthologue transcripts including 15,053 human unique transcripts hits and 14,892 different bovine unique transcripts hits (SI1 Table 3). These transcripts represented 11,533 human and 10,774 bovine genes. Additionally we also translated the transcript sequences into protein sequences (25,520) and aligned them with BLASTp against the human and bovine proteome. On protein level, 20,599 proteins were identified to be human (12,512) or bovine (13,050) orthologues or homologs. These numbers corresponded to 10,123 human and 9,911 bovine protein-coding genes. The numbers for the other assemblies are shown in SI1 Table 3.

SI1 Table 3. BLASTn and BLASTp result for all four different assemblies (BTA = *bos taurus*; HSA = *homo sapiens*).

**assembly**	**# of transcripts TPM>2**	**# of detected peptides**	**orth. protein HSA or BTA**	**orth. transcript HSA or BTA**	**HSA orth. transcript**	**BTA orth. transcript**	**HSA orth. genes transcript**	**BTA orth. genes transcript**	**HSA orth. protein**	**BTA orth. protein**	**HSA orth. genes protein**	**BTA orth. genes protein**
**embryo**	46,652	10,159	7618	16,098	8771	8490	7568	7009	6082	6237	5354	5259
**endo-metrium**	50,673	13,430	9850	20,535	10,298	9842	8665	7943	7725	7950	6678	6572
**mixed tissue**	44,338	15,563	11,713	18,644	10,937	10,381	9078	8378	9255	9443	8063	7908
**joined**	63,935	25,520	20,599	33,980	15,053	14,892	11,533	10,774	12,512	13,050	10,123	9911

BLASTn of all assemblies against each other

Additionally, all transcriptome assemblies were compared by aligning the corresponding transcripts. Only the best hit was reported and all hits were filtered as explained in the methods section. The diagram in Figure S7 shows that all individual transcriptome assemblies had a relatively high percentage of tissue-specific transcripts, with the highest percentage for embryos tissue. The comparison to the joined assembly showed that part of the transcripts of the individual assemblies were lost during joining, with the highest percentage for the mixed tissue assembly. The highest overlap of transcripts was found for the MT in comparison to the other individual assemblies.

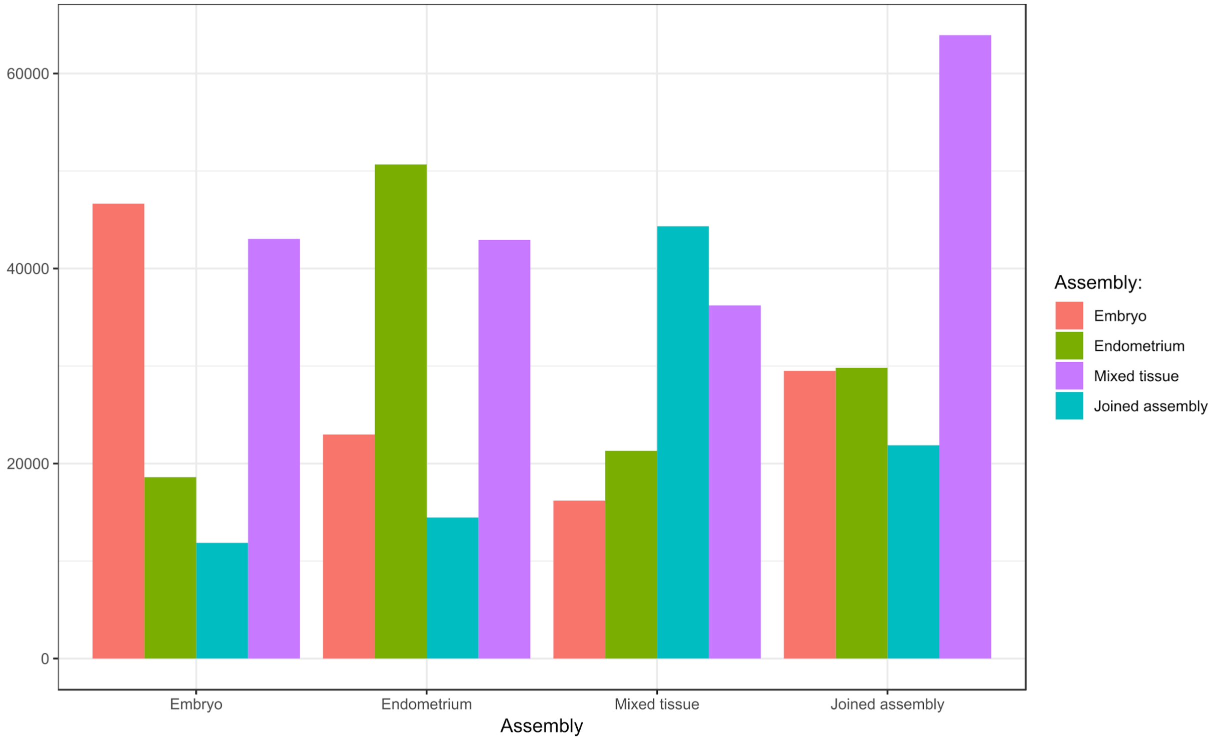

Figure S7. BLASTn transcript overlap between the transcriptome assemblies.

Supplementary information 2

Sequencing read statistics

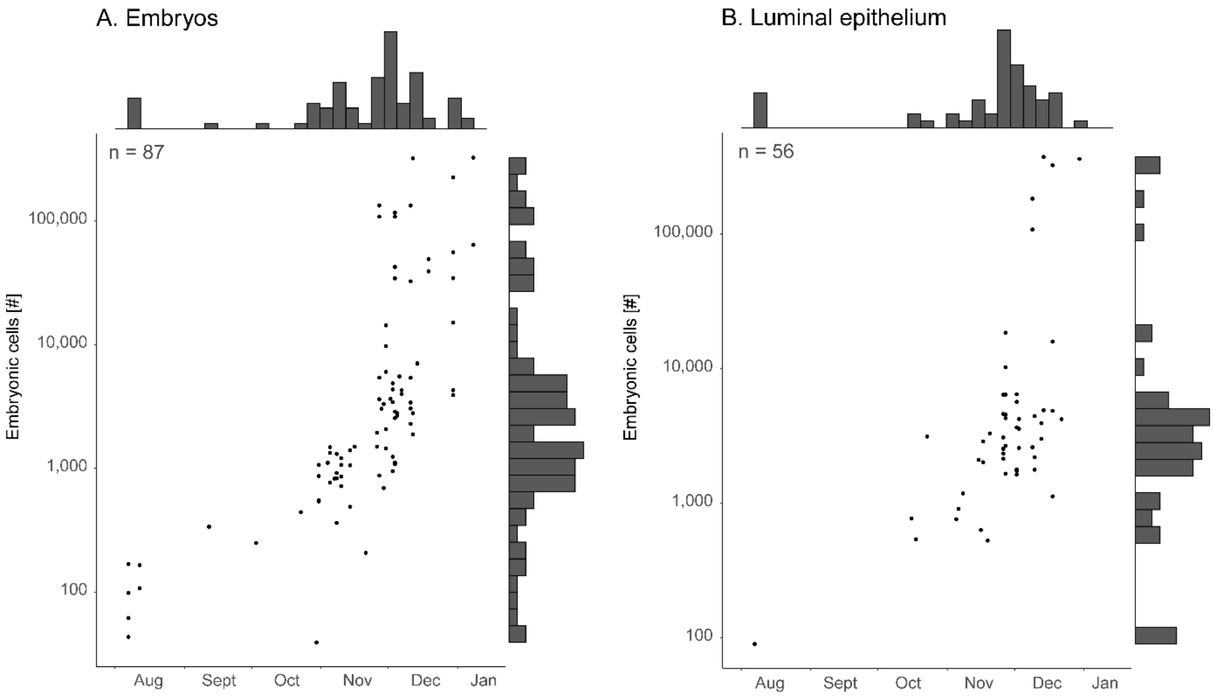

Figure S8: Embryonic development displayed as number of embryonic cells over sampling date with histograms indicating the number of collected embryos. **A.** Embryos and **B.** Luminal epithelium.

RNA sequencing was performed on 87 single embryos covering the pre-implantation embryo development. A total of 4175 million raw reads was obtained after RNA sequencing, with a duplication rate of 82 ± 7 % (mean ± SD) and a % GC of 55 ± 3 %. After the removal of low-quality reads and PCR duplicates, 1092 million reads were used for further analysis. The duplication rate in the cleaned sequences was 8 ± 2 % (mean ± SD) and the GC% was 56 ± 3 %.

RNA sequencing was performed on 56 LE samples covering the pre-implantation embryo development. A total of 1524 million raw reads was obtained after RNA sequencing, with a duplication rate of 69 ± 6 % (mean ± SD) and a % GC of 49 ± 2 %. After removal of low-quality reads and PCR duplicates, 419 million reads were used for further analysis. The duplication rate in the cleaned sequences was 3 ± 2 % (mean ± SD) and the GC% was 47 ± 2 %.

The DET were annotated against the bovine and human genome and transcript variants were aggregated to obtain single expression values for each gene symbol to allow functional analysis. A total of 9,546 DEG in the embryos and 1,833 DEG in the LE were used for functional analysis with the QIAGEN Ingenuity Pathway Analysis (IPA) software.

Supplementary Information 3

RNA-seq analysis pipeline to retrieve the *de novo* assembly

All FastQ files were checked on quality using FastQC. This checking was conducted after all sequence improvements. The first tool in the pipeline was the Rcorrector tool, to correct for random sequencing errors in Illumina RNA-seq reads, which was wrapped for Galaxy. All FastQ files were cleared from PCR duplicates by using FastUniq [1], followed by quality filtering which was done by running "TrimGalore!" to remove the adapter sequence and trim on sequence quality (min quality score of 28) with a minimum sequence length of 70 bp. After these basic sequence improvements, the *de novo* assembler Trinity (version 2.6.6) was used to construct the transcriptome assembly. The PacBio reads were used as a parameter (--long-reads) to improve the assembly. Contigs shorter than 200 nt were discarded by default from the assembly by Trinity. Transcriptome assemblies were performed at the high-performance computing facilities of ETH Zurich, the "Euler cluster", a part of the Swiss National Supercomputing Center in Lugano Switzerland (https://www.cscs.ch). The PacBio reads were assembled beforehand with Pacific Biosciences SMRT® (version v2.3.0) using the transcriptome assembler tool IsoSeq1 (https://github.com/PacificBiosciences/IsoSeq_SA3nUP/wiki). Three different assemblies were generated for the elongated embryo, endometrium, and both tissues combined by using the assembly concatenation tool [2]. These three high-quality consensus FASTA assemblies were used for the three appropriated Illumina sequenced tissues as a guideline. TransRate was used to evaluate and benchmark the generated Trinity transcriptomes and to map back all Illumina paired-end reads. This mapping was performed with Salmon [3], running with quasimappings ideal for fast and accurate short-read mapping to transcriptomes by using k-mer. TransRate also uses several methods to filter, error correct, and optimize all transcripts, such as unsupported insertions, fragmentation, and redundancy of transcripts. An R based pipeline was run to filter the assembly FASTA files on two transcripts per million (TPM):

$$TPM = \frac{\frac{counts}{length}}{\sum \frac{counts}{lenght} 10^6}$$

In order to reduce the number of low abundant assembly artefacts, the following statistical values were calculated for a better visualization and validation of the transcriptomes: N50, average, median, maximum and minimum length. For the TPM per transcript, the average and median of all TPM filter steps were calculated, as well as the total number of sequences left according to the TMP filter steps. The N50 is computed in such a way that first all sequences are sorted by length and concatenated as one long string. The N50 is exactly the sequence length of the sequence in the middle (N50 meaning 50%). That means that the N50 is the size of the smallest of all the large transcripts covering 50% of the transcriptome [4]. The N50 is quite insensitive to a large number of small transcripts, but considered to be a better quality estimate of the assembly than the mean or median of all transcripts lengths (http://resources.qiagenbioinformatics.com//whitepapers/White_paper_on_de_novo_assembly_4.pdf). Additionally, all single generated transcriptomes were joined into one final assembly using transfuse (https://github.com/cboursnell/transfuse). Transfuse has two important inputs, a list of all assemblies in FASTA format and a list of all paired-end reads. The output is a single high-quality transcriptome additionally validated with TransRate within the pipeline.

Quantitative LC-MS/MS analysis of uterine fluid amino acids

Thirty µl of uterine fluid was dissolved in 500 µl ice-cold methanol containing an internal standard mixture of 15 deuterated AA, 12 deuterated acylcarnitines and deuterated free carnitine. Samples were dried after centrifuging for 10 minutes at 10°C. Derivatisation of metabolites to their butyl esters was done by incubating the samples in a mixture of 95% n-butanol and 5% acetylchloride (v/v) at 60°C for 15 minutes at 600 rpm (Eppendorf Thermomixer Comfort; Eppendorf, Hamburg, Germany) [5]. The samples were dried and reconstituted in 200 µl of methanol/water/formic acid (70/30/0.1% v/v). The analysis was performed on a triple quadrupole QTRAP 5500 LC-MS/MS system operating in positive ESI mode (AB Sciex, Framingham, MA) equipped with a 1200 series binary pump (Agilent, Santa Clara, CA) and coupled to an HTC pal auto-sampler (CTC Analytics, Zwingen, Switzerland). Chromatographic separation was achieved using a Zorbax Eclipse XDB-C18 column (length 150 mm, internal diameter 3.0 mm, particle size 3.5 µm; Agilent). Analytes were measured in scheduled multiple reaction monitoring. For quantification of AA, a 10-point calibration was performed using a mixture containing all AA in the measurement (A9906 AA standards, SigmaAldrich, Taufkirchen, Germany).

Insulin and insulin growth factor 1 quantification

Before running the assay, inactivated and insulin-free foetal bovine serum (FBS) was prepared. FBS was heated at 55°C for 1 hour and filtered with a 0.22 µm membrane. Insulin was removed using 50 mg/ml of active charcoal at 4°C for 30 min. Three 20 min centrifugations at 4000 rpm were used to remove the charcoal. The FBS was filtered again with a 0.22 µm membrane and aliquots were stored at -20°C. Standard samples of decreasing insulin concentrations were prepared in inactivated and insulin-free FBS. Plasma samples were thawed and maintained at 4°C during the time of assay. Four microliter samples were distributed in duplicate in opaque 96 well plates (½ AreaPlateTM, PerkinElmer, USA). A 4 µL mix of anti-insulin antibody (1 nM final concentration) and acceptor beads (10 µg/ml final concentration) was then added. After 1 hour incubation at room temperature in the dark, 32 µL of streptavidin-coated donor beads (40 µg/ml final concentration) were added. Plates were analysed after 30 min incubation in the dark at room temperature, using the Enspire® reader and Manager software (PerkinElmer, USA).

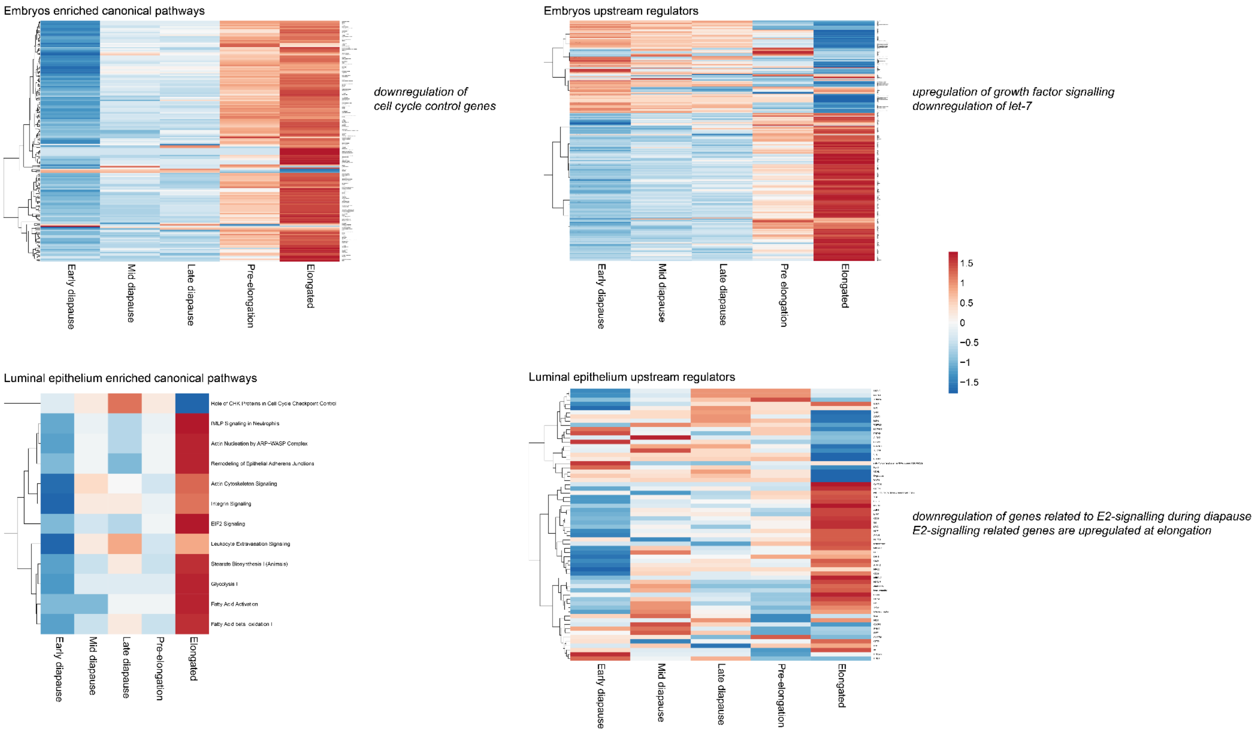

Figure S9. Heatmaps displaying the enriched canonical pathways and upstream regulators in the embryos and luminal epithelium. All pathways and upstream regulators with a z-score cut-off of >2 in one of the analyses and p-value of <0.05 are included.

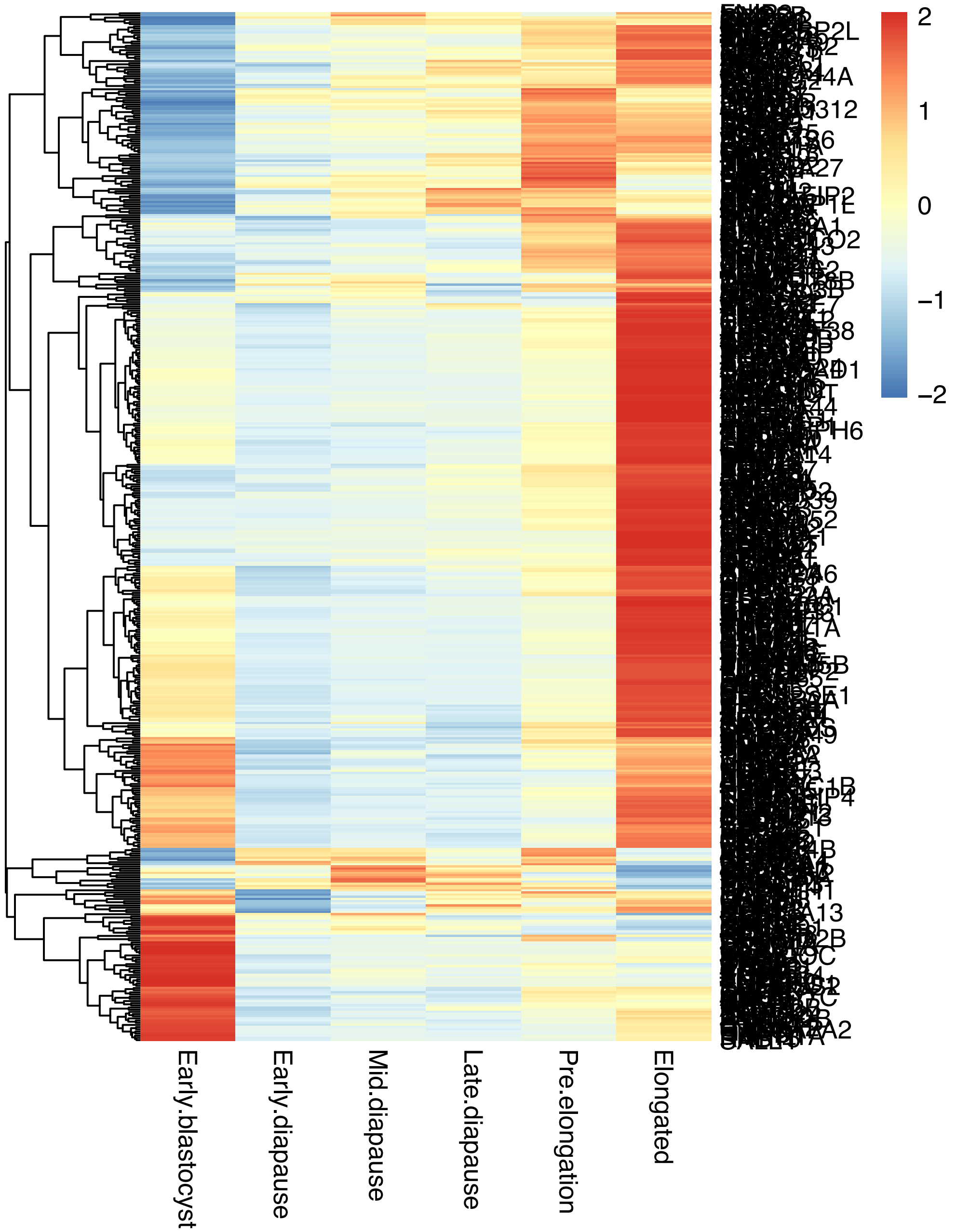

Figure S10. Heatmap displaying the z-scores of let-7 and let-7a-5p targets at defined developmental stages. Expression of single target genes can be found in Dataset S1.

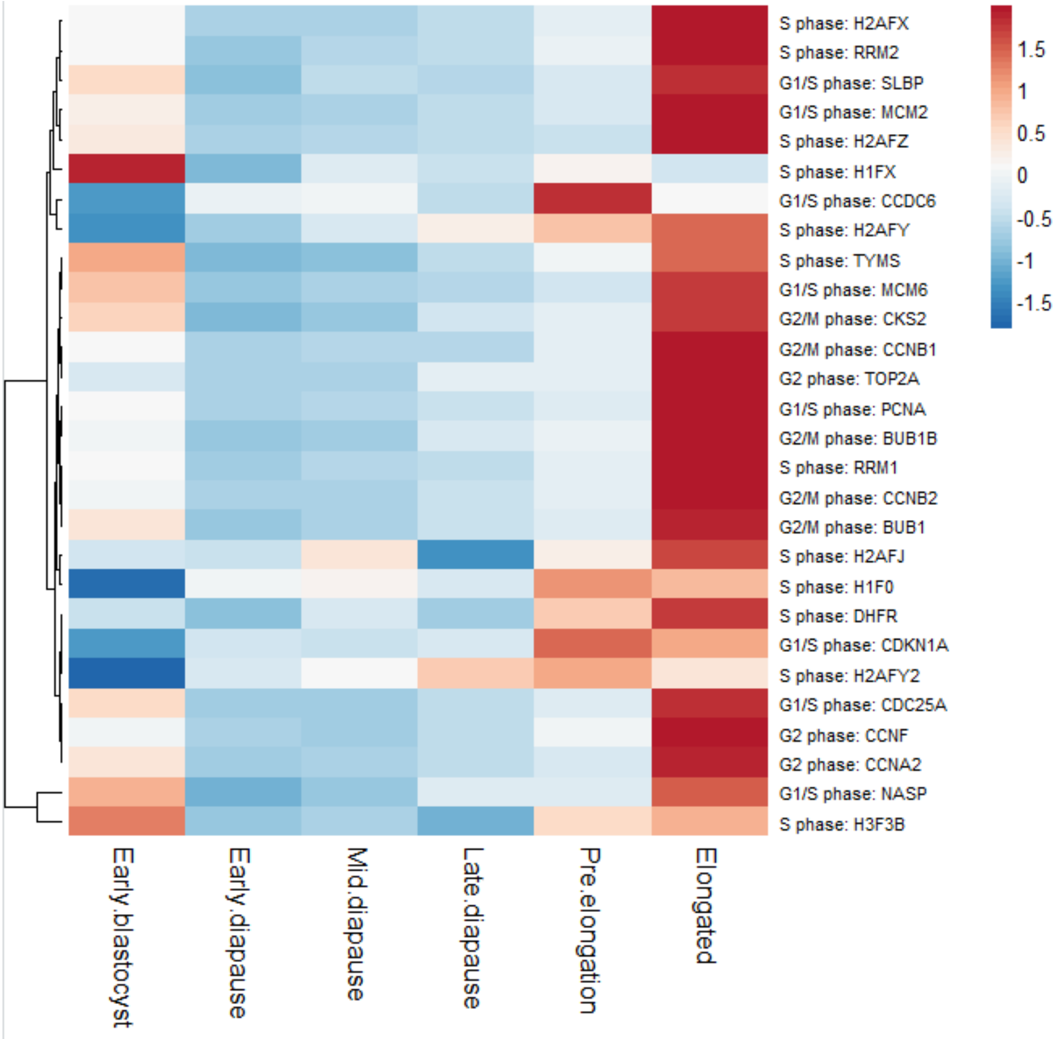

Figure S11. Heatmap displaying the cell cycle phase-specific genes as previously described by Whitfield et al. (2002) and their expression at defined developmental stages.

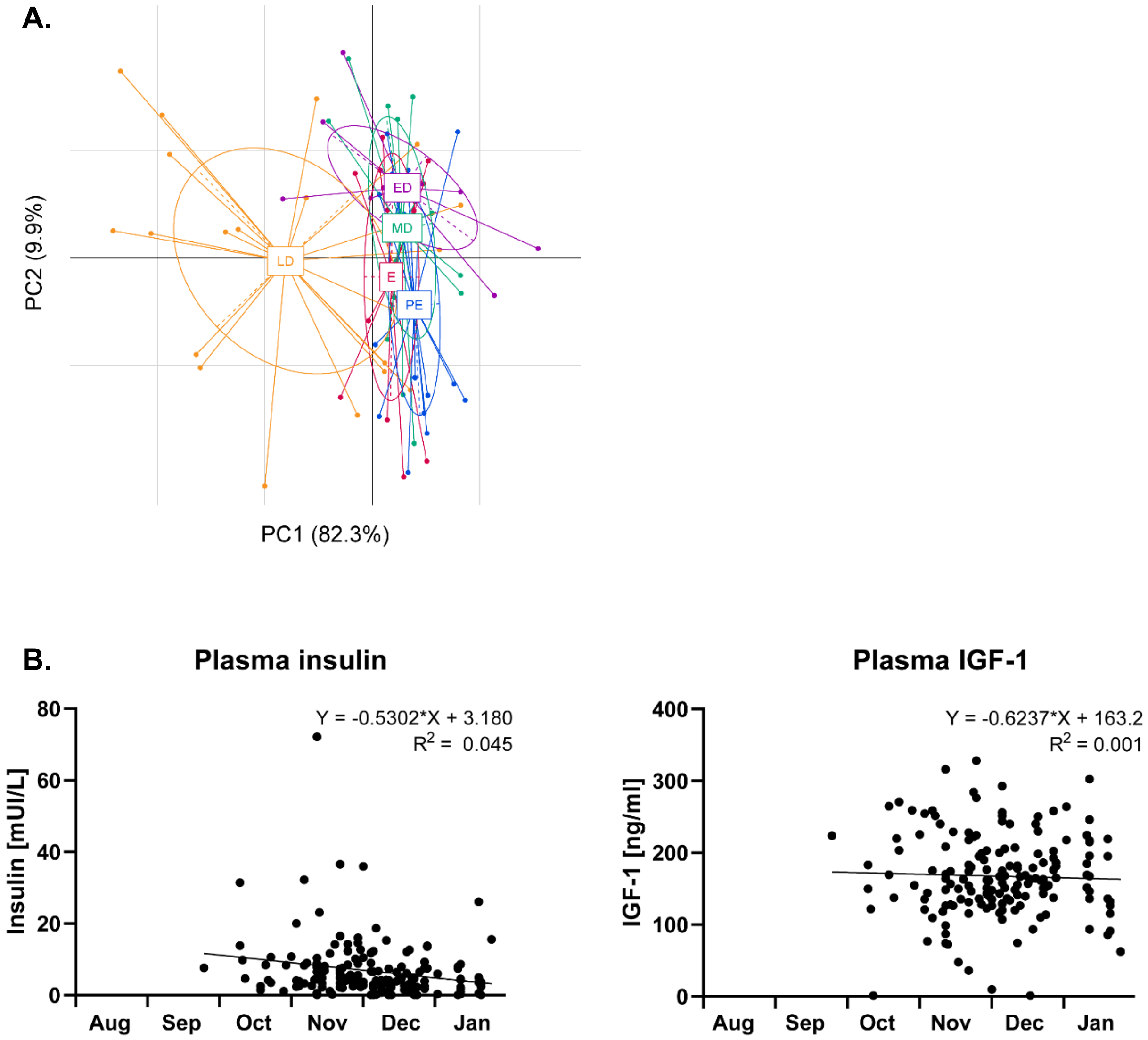

A.
ED
MD
LD
E
PE
PC2 (9.9%)
PC1 (82.3%)
B.
Plasma insulin
Y = -0.5302*X + 3.180
R2 = 0.045
Insulin [mUI/L]
80
60
40
20
0
Aug
Sep
Oct
Nov
Dec
Jan
Plasma IGF-1
Y = -0.6237*X + 163.2
R2 = 0.001
IGF-1 [ng/ml]
400
300
200
100
0
Aug
Sep
Oct
Nov
Dec
Jan

C. Luminal epithelium melatonin target genes

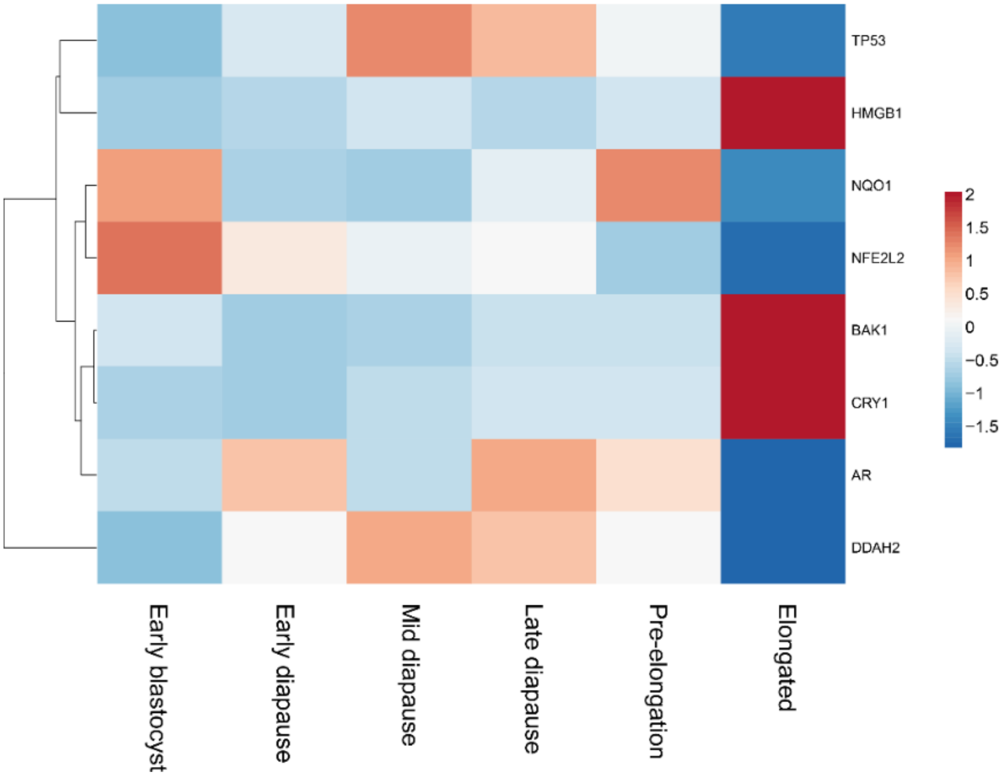

D. Luminal epithelium prolactin responsive genes

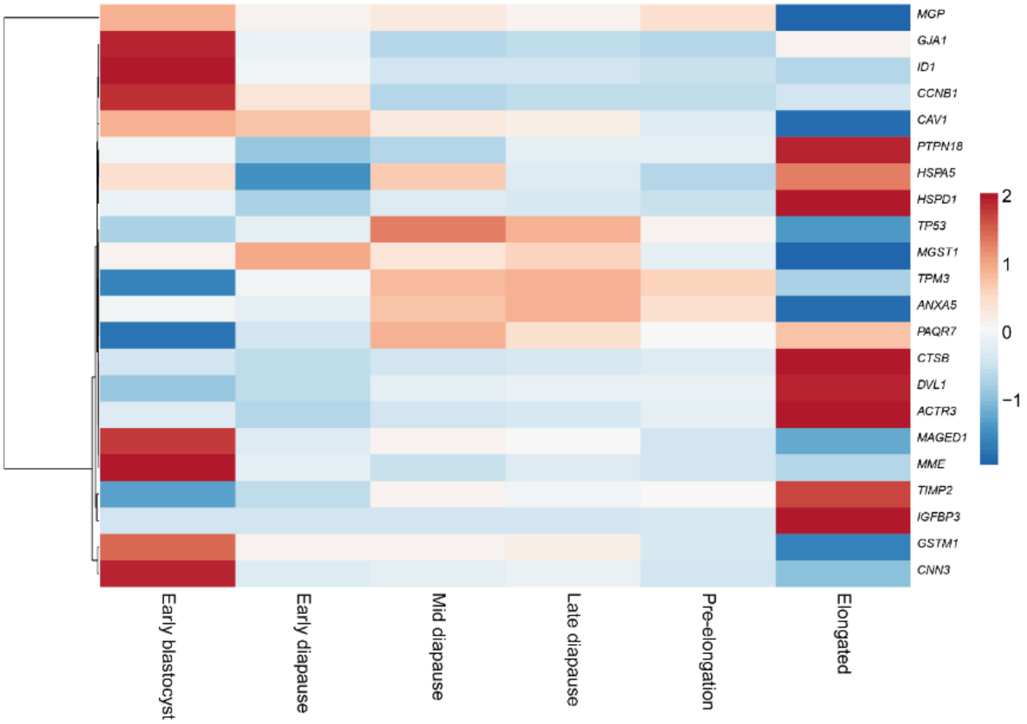

E.

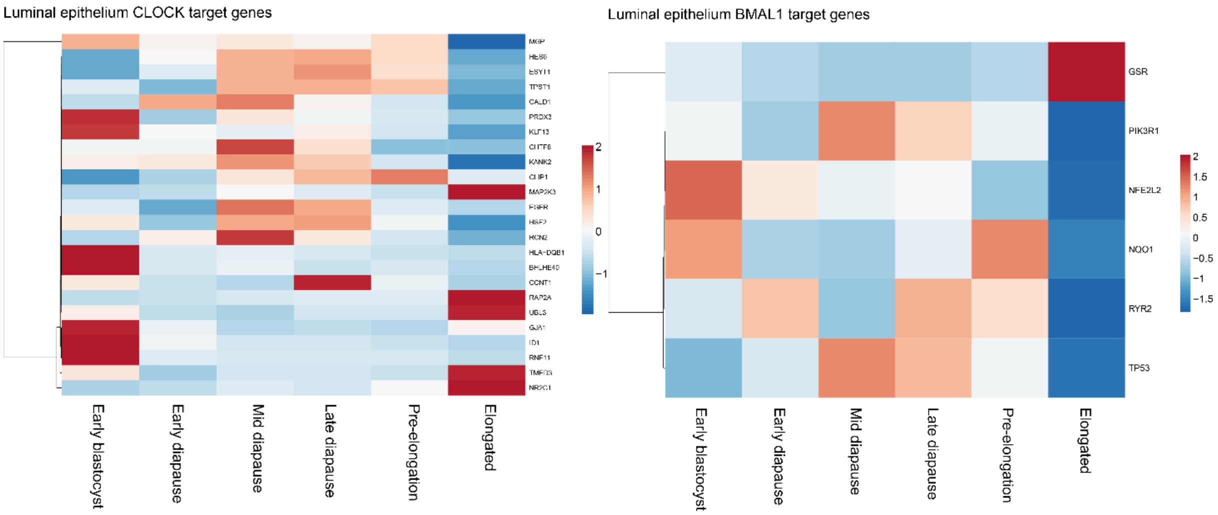

Figure S12. A. Between group analyses of the plasma amino acids in 72 animals covering the pre-implantation period. ED = early diapause, MD = mid diapause, LD = late diapause, PE = pre-elongation, and E = elongated. **B.** Plasma levels of insulin and IGF-1 according to sampling date. **C.** Heatmap displaying the expression of melatonin target genes in the luminal epithelium. **D.** Heatmap displaying the expression of prolactin responsive genes in the luminal epithelium. **E.** Heatmaps displaying the expression of circadian genes including the CLOCK and BMAL1 target genes in the luminal epithelium.

Dataset S1 (separate file). Enriched canonical pathways and upstream regulators in the embryos and luminal epithelium. Let-7 and Let-7a-5p target gene expression in the embryos.

Dataset S2 (separate file). Absolute quantification data of the amino acids in the uterine fluid in µM.

Dataset S3 (separate file). Roe deer sample list with the conducted analyses per sample.

Dataset S4 (separate file). qPCR and RNASeq data luminal epithelium.

SI References

1. Xu, H., et al., *FastUniq: A Fast De Novo Duplicates Removal Tool for Paired Short Reads.* PLOS ONE, 2012. **7**(12): p. e52249.

2. Nakasugi, K., et al., *Combining transcriptome assemblies from multiple de novo assemblers in the allo-tetraploid plant Nicotiana benthamiana.* PLoS One, 2014. **9**(3): p. e91776.

3. Patro, R., et al., *Salmon provides fast and bias-aware quantification of transcript expression.* Nature Methods, 2017. **14**: p. 417.

4. Miller, J.R., S. Koren, and G. Sutton, *Assembly algorithms for next-generation sequencing data.* Genomics, 2010. **95**(6): p. 315-27.

5. Gucciardi, A., et al., *A rapid UPLC-MS/MS method for simultaneous separation of 48 acylcarnitines in dried blood spots and plasma useful as a second-tier test for expanded newborn screening.* Anal Bioanal Chem, 2012. **404**(3): p. 741-51.
